# High specificity and tight spatial restriction of self-biotinylation by DNA and RNA G-Quadruplexes complexed *in vitro* and *in vivo* with Heme

**DOI:** 10.1093/nar/gkaa281

**Published:** 2020-04-24

**Authors:** Prince Kumar Lat, Kun Liu, Dev N Kumar, Kenneth K L Wong, Esther M Verheyen, Dipankar Sen

**Affiliations:** 1 Department of Molecular Biology and Biochemistry, Simon Fraser University, Burnaby, British Columbia V5A 1S6, Canada; 2 Department of Chemistry, Simon Fraser University, Burnaby, British Columbia V5A 1S6, Canada

## Abstract

Guanine-rich, single-stranded DNAs and RNAs that fold to G-quadruplexes (GQs) are able to complex tightly with heme and display strongly enhanced peroxidase activity. Phenolic compounds are particularly good substrates for these oxidative DNAzymes and ribozymes; we recently showed that the use of biotin-tyramide as substrate can lead to efficient GQ self-biotinylation. Such biotinylated GQs are amenable to polymerase chain reaction amplification and should be useful for a relatively non-perturbative investigation of GQs as well as GQ–heme complexes within living cells. Here, we report that in mixed solutions of GQ and duplex DNA *in vitro*, GQ biotinylation is specifically >10^4^-fold that of the duplex, even in highly concentrated DNA gels; that a three-quartet GQ is tagged by up to four biotins, whose attachment occurs more or less uniformly along the GQ but doesn’t extend significantly into a duplex appended to the GQ. This self-biotinylation can be modulated or even abolished in the presence of strong GQ ligands that compete with heme. Finally, we report strong evidence for the successful use of this methodology for labeling DNA and RNA within live, freshly dissected *Drosophila* larval salivary glands.

## INTRODUCTION

Single-stranded DNAs (ssDNAs) and RNAs are capable of forming a number of non-canonical secondary structures, including G-quadruplexes (GQs) (1–3), i -motif (4), R-loops (5), triplexes (6) and other triplex-like hybrids (7). Guanine-rich nucleic acids have a strong propensity to fold, under physiological temperature and solution conditions, into GQs, structurally polymorphic quadruple helices that can form either in intramolecular or intermolecular fashion, with a diversity of topologies and strand orientations. GQs are held together by Hoogsteen-bonded and π-stacked guanine base-quartets (1). A diversity of biological role(s) has been proposed for GQ DNAs and RNAs, and they continue to be the subject of intensive computational and experimental investigations (8–10).

To date, a large number of distinct experimental approaches have been used to search for GQs within living cells (8–11). Extensive use has been made of strongly GQ-binding small molecules (‘GQ-ligands’) to stabilize and/or pull-down intracellular DNA and RNA GQs (11–15). One caveat with regard to the use of extrinsic GQ-ligands to probe living cells is the possibility that such ligands may perturb the equilibrium of non-GQ but GQ-capable DNA or RNAs toward forming GQs. Yet GQ-ligands do not have to be either extrinsic or synthetic compounds. We have shown that the ubiquitous cellular cofactor, hemin [ferric heme or Fe(III)-protoporphyrin IX], present in all cells, is itself a GQ-ligand, complexing tightly with both RNA and DNA GQs (*K*_d_ values in the 10–500 nM range (16–23)). Hemin binds by end-stacking on the terminal G-quartets of GQs; it has been shown that parallel-stranded GQs bind hemin more strongly than antiparallel-stranded ones (24). *In vitro*, such GQ–hemin complexes, in the presence of hydrogen peroxide (or gaseous oxygen in the presence of a reductant, such as ascorbate), show robust, DNA/RNA-enhanced oxidative catalysis of both 1-electron (peroxidase) and 2-electron (peroxygenase/oxygenase) oxidation reactions (16–24). Since the discovery of the complexation of hemin by GQs in 1998, this interaction has been intensively investigated, and no other fold of DNA or RNA (with the exception of the non-physiological isoguanine pentaplexes (25)) has been reported either to complex hemin or to activate it toward oxidative activity (24,26). In a most interesting development, experimental evidence has recently been reported to validate earlier conjectures (20,21) that cellular RNA/DNA GQs naturally sequester intracellular hemin (27).

Interestingly, GQ–hemin complexes are able to oxidize phenolic substrates, such as tyrosine, tyramine, and substituted tyramides, faster than does horseradish peroxidase (HRP) (28). We recently reported that *in vitro*, treating GQ–hemin complexes with hydrogen peroxide and the commercially available reagent biotin tyramide (BT), leads to efficient covalent biotinylation of the GQs themselves (29). This can be conveniently monitored by complexing the biotinylated GQs with streptavidin (StAv) and observation of concomitant electrophoretic mobility retardation of these GQ•biotin-StAV complexes in polyacrylamide gels. By contrast, initial experiments with DNA *duplexes* co-dissolved with GQ–hemin complexes showed no trace of duplex biotinylation and, correspondingly, no StAv-retarded gel mobility of the duplexes. That the StAv-shifted DNAs were indeed biotinylated had been confirmed using MALDI-TOF mass spectrometry analysis in our earlier study (29).

Herein, we report a thorough investigation of the extent, specificity and spatial limits of the GQ self-biotinylation reaction as it occurs *in vitro*. We then go on to provide convincing results using this methodology for biotinylation of nucleic acids within freshly dissected, live *Drosophila* tissue.

## MATERIALS AND METHODS

### Chemicals and DNA oligonucleotides

SAvPhire Monomeric StAv (‘monoavidin’) was purchased from Sigma-Aldrich. Trolox ((±)-6-Hydroxy-2,5,7,8-tetramethylchromane-2-carboxylic acid) was from Sigma-Aldrich. Luminol (Pierce ECL Western Blotting Substrate) and StAv-HRP were from ThermoFisher Scientific.

All DNA and RNA oligonucleotides were purchased from the Core DNA Services Inc. (Calgary, Canada). The oligonucleotides were first treated for 30 min with 10% v/v aqueous piperidine at 90°C to cleave DNA strands containing synthesis-related chemical lesions, lyophilized, and size purified in 8–12% denaturing gels. The DNA was then ethanol precipitated and dissolved in TE buffer (10 mM Tris, pH 7.4, 0.1 mM ethylenediaminetetraacetic acid) to make stock solutions of desired concentrations. All DNA oligonucleotides used in this study were gel-purified by size. As required, oligonucleotides were 5′-labeled with ^32^P using γ-^32^P adenosine triphosphate (ATP) and a standard kinasing protocol, then polyacrylamide gel electrophoresis-purified. The DNA and RNA oligonucleotides used in this study are shown in Table 1.

**Table 1.** DNA and RNA oligonucleotides used in this work

***CatG4***	5′-TGG GTA GGG CGG GTT GGG AAA (21 nt)
***CatG4-ext***	5′-ACA TAG CTG ACT GGC TTG ATT TTG GGT AGG GCG GGT TGG GAA ATA TCG AAT TCT CAG CCT ACA CTG CAG TAC TAG TAC ATA TCA (84 nt)
***dsDNA Watson strand***	5′-CCC ATT CTA TCA TCA ACG GGT ACA AAC GAG TCC TGG CCT TGT CTG TGG AGA CGG ATT ACA CCT TCC CAC TTG CTG (75 nt)
***dsDNA Crick strand***	5′-CAG CAA GTG GGA AGG TGT AAT CCG TCT CCA CAG ACA AGG CCA GGA CTC GTT TGT ACC CGT TGA TGA TAG AAT GGG (75 nt)
***CatG4-T7***	5′-TGG GTA GGG CGG GTT GGG AAA TAT CCT ATA GTG AGT CGT ATT AAA G (46 nt)
***ssDNA/BLD:***	5′-CTT TAA TAC GAC TCA CTA TAG G (22 nt)
***CatG4-ext2***	5′-TGG GTA GGG CGG GTT GGG AAA TAT TTT AGC TCA CGA GAC GCT CCC ATA GTG ACC TAT AGT GAG TCG TAT TAA AGT CCG GAG GAC TGT CCT CCG GC (95 nt)
***Comp 1***	5′-TCA CTA TGG GAG CGT CTC GTG AGC TAA A (28 nt)
***Comp 3***	5′-GCC GGA GGA CAG TCC TCC GGA (21 nt)
***MYC***	5′- TGA GGG TGG GTA GGG TGG GTA A (22 nt)
***KIT1***	5′- AGG GAG GGC GCT GGG AGG AGG G (22 nt)
***KIT2***	5′- CGG GCG GGC GCG AGG GAG GGG (21 nt)
***SPB1***	5′- GGC GAG GAG GGG CGT GGC CGG C (22 nt)
***TBA***	5′- GGT TGG TGT GGT TGG (15 nt)
***hTELO***	5′- GGT TAG GGT TAG GGT TAG GGT TAG GGT TAG (30 nt)
***rBCL2 (RNA)***	5′- r(AGG GGG CCG UGG GGU GGG AGC UGG GG) (26 nt)
***rNRAS (RNA)***	5′- r(AGG GAG GGG CGG GUC UGG G) (19 nt)
***r(ssRNA)***	5′- r(UGA UUA GGA UCU GCC AAC CGU G) (22nt)

### Standard GQ biotinylation reaction

GQ DNA stock solution was diluted appropriately into Q Buffer (40 mM HEPES, pH 8.0, 20 mM potassium chloride, 1% dimethylformamide and 0.05% Triton X-100), and then heat-denatured at 100°C for 3 min, followed by cooling to 22°C by directly transferring the reaction tubes from boiling water bath to that temperature. Hemin was added to the folded DNA, and the solution rested for 10 min to assist heme–GQ complex formation. BT and H_2_O_2_ were then added to initiate the biotinylation reaction for various time points. The reactions were quenched with the addition of 10 U bovine liver catalase (Sigma-Aldrich).

### Native gel electrophoresis and gel data analysis

DNA biotinylation was verified using a StAv gel shift assay in 7.5% non-denaturing/native polyacrylamide gels (acrylamide:bis = 29:1) run in 50 mM Tris borate (TBE) buffer. Biotinylated DNA was mixed with StAv in aqueous solution prior to loading in native gels run at 22°C with efficient cooling. Imaging and densitometry analysis of ^32^P-labeled gel bands were done using a Typhoon 9410 Phosphorimager (Amersham Biosciences). Quantitation was carried out using ImageQuant 5.2 software (Amersham).

### Determination of biotinylation stoichiometry using ‘monoavidin’

For determining biotinylation stoichiometries, 1 μM ‘CatG4’ DNA was denatured at 100°C and refolded in Q buffer for 30 min at 22°C. Hemin was added to 5 μM, and the mixture allowed to incubate for a further 10 min. A total of 500 μM BT and 1 mM H_2_O_2_ were now added to initiate the biotinylation reaction, which proceeded for 30 min. The reaction was quenched by the addition of 10 U of catalase. The treated DNA was recovered by ethanol precipitation and mixed with aqueous solutions of either StAv or Monomeric Avidin ('Monoavidin' or MAv). The biotinyl-DNA/protein complexes were then run in a native polyacrylamide gel run at 4°C.

### Biotinylation competition experiments

For dilute solution competition experiments, ‘CatG4-ext’ (0.01 μM, either 5′-^32^P-labeled or not, depending on the experiment) was heat-denatured for 3 min at 100°C. Following 5 min of incubation in Q buffer at 22°C, heme was added to 5 μM and rested 10 min to assist complexation. Duplex DNA (dsDNA, made from the annealing of 100 μM each of ‘dsDNA Watson Strand’ and ‘dsDNA Crick Strand’, 5′-^32^P-labeled or not, depending on the experiment), or ssDNA (100 μM, 5′-^32^P-labeled or not) was now added. The final reaction buffer (QD Buffer: 40 mM HEPES, 40 mM Tris, pH 8.0, 20 mM KCl, 26 mM MgCl_2_, 2.5 mM spermidine-Cl_3_, 1% dimethylformamide and 0.05% Triton X-100) contained both potassium to stabilize the GQ and magnesium and spermidine to stabilize the duplex. The total DNA/hemin mixture was equilibrated at 22°C for 30 min, following which, BT (to 500 μM) and H_2_O_2_ (to 1 mM) were added and the peroxidase reaction allowed to proceed for 30 min. The reaction was quenched using 10 U of bovine liver catalase (Sigma). The treated DNA mixture was recovered by ethanol precipitation, and the 70% ethanol-washed DNA pellet was dissolved in an aqueous solution of 25 μg (45 μM) StAv prior to running in a 7.5% native polyacrylamide gel run in 50 mM TBE buffer.

For the labeling competition experiments carried out at ultra-high overall DNA concentrations, the procedure was the same as above, with the exception that sheared duplex salmon sperm DNA (Sigma) was added to 17.5 mg/ml to the dsDNA mixture with hemin/GQ prior to 30-min equilibration at 22°C and initiation of the peroxidase/biotinylation reaction.

### Determination of distribution of biotinylation within a GQ

CatG4 (1 μM) and other singly riboside-modified oligonucleotides, ‘CatG4_R_x_’ (*x* = 1, 2, 3) (1 μM), were denatured for 3 min at 100°C and refolded in Q Buffer for 30 min at RT. A total of 5 μM heme was then added and the solution equilibrated for 10 min. Following this, 500 μM BT and 1 mM H_2_O_2_ were added to initiate the reaction, which proceeded for 30 min at 22°C prior to quenching by addition of catalase. The DNA was then ethanol precipitated, dissolved in TE buffer, and the solution was divided into two halves. To cleave a given ‘CatG4_R_x_’ at its internal ribonucleotide one half of the DNA solution was treated with 0.25 M NaOH at 90°C for 5 min; following which, the solution was neutralized with equimolar HCl. The NaOH-cleaved strands were resolved and purified from a 10% denaturing gel. Biotinylated DNA species were identified via treatment with StAv and subsequent analysis in 7.5% native gels.

### Determination of the radius of active biotinylation

A total of 1 μM ‘CatG4-T7’ and 1 μM ‘ssDNA’ (both 5′-^32^P-labeled with γ-^32^P ATP) were pre-denatured separately for 3 min at 100°C in TE buffer. They were mixed together in QD Buffer and allowed to anneal by slow cooling (from 100°C to 20°C at a rate of 7.5°C/min) in a Thermocycler. The solution was now made up to 5 μM heme and allowed to equilibrate for 10 min. A total of 500 μM BT and 1 mM H_2_O_2_ were added and the biotinylation reaction allowed to proceed for 30 min. The reaction was quenched with catalase and the two component DNA strands (‘CatG4-T7’ and ‘ssDNA’) were separated and purified in an initial 8% denaturing gel. The recovered DNA, ethanol precipitated, was redissolved and treated with StAv prior to running in a 7.5% native gel.

For investigation of longer-range biotinylation along the length of GQ-duplex composites, three short oligonucleotides, ‘Comp-1’, ‘ssDNA’ and ‘Comp-3’—each complementary to a different stretch of the oligonucleotide ‘CatG4-ext2’—were annealed together at 1 μM concentration each to ‘CatG4-ext2’ (with only one of the shorter oligonucleotide ^32^P-labeled at a time). The subsequent procedure was carried out as described for the complex formed between ‘CatG4-T7’ and ‘ssDNA’, above.

### Competition with GQ-binding ligands

A total of 1 μM ‘CatG4-ext’ was denatured for 3 min at 100°C and refolded in Q buffer for 30 min at 22°C. It was then made up to 5 μM hemin and rested for 10 min. Different concentrations (0–200 μM) of a given GQ-binding ligand was added, and the solution equilibrated further for 10 min. Following this, the solution was made up to 500 μM BT and 1 mM H_2_O_2_ to initiate the biotinylation reaction, which proceeded for 30 min at 22°C prior to quenching by the addition of catalase. The DNA was then recovered by ethanol precipitation and co-dissolved with StAv prior to running on native gels for analysis.

### *In vivo* biotinylation reaction and biotin blotting

*Drosophila melanogaster* salivary glands were dissected into PBS buffer (phosphate buffered saline, pH 7.4) and incubated with 50 μM hemin, 3 mM BT for 15 min, following which it was given a pulse of 10 mM H_2_O_2_ for 2–3 min (H_2_O_2_ was added and the solution containing the tissue shaken gently on a shaker for 2–3 min). Following this treatment, the glands were washed twice with PBS buffer containing radical quenchers and peroxidase inhibitors (5 mM Trolox, 10 mM sodium azide, 10 mM sodium ascorbate). Genomic DNA as well as total RNA were extracted from treated salivary glands using a ‘DNeasy Blood & Tissue Kit’ (Qiagen) and an ‘RNeasy Mini Kit (Qiagen), respectively, per the manufacturer's protocols. To take extra precautions to ensure the purity of the DNA extractions, DNA as extracted using the kit was given further treatments of RNase and proteinase as follows: first, the DNA was incubated with RNase A at 37°C for 1 h, after which it was incubated with Proteinase K at 50°C for 12–14 h. Following the above treatment, the DNA solution was extracted twice with phenol: chloroform: isoamyl alcohol (25:24:1), and then once with chloroform alone. The treated DNA was then recovered by way of two successive ethanol precipitations, followed by a 70% ethanol wash. The purified DNA pellet was then dissolved in TE buffer for further downstream analysis.

A total of 2–5 μL of purified total RNA or genomic DNA was then spotted onto a Hybond N+ membrane using a standard dot blot transfer apparatus and were crosslinked to the membrane using a UV Crosslinker (a UV Stratalinker 2400) using 254 nm light source, at a flux of 120 000 μJ for 30 s. The membrane was then treated with Blocking Buffer [1 × Tris Buffered Saline (TBS) containing 5% bovine serum albumin, 0.5% sodium dodecyl sulphate (SDS), 0.1% Ficoll, 50 μg/ml salmon Sperm DNA and 0.5 ug/ml poly d-IC DNA] for 90 min at 22°C. The blocking buffer was discarded and the membrane swirled overnight at 4°C with fresh blocking buffer of the same composition but containing, additionally, 1:10 000 diluted StAv-HRP. The next day, the StAv-HRP containing buffer was discarded and the membrane thoroughly washed, with swirling in Washing Buffer (1 × TBS containing 0.5% SDS) twice for 45 min each at 22°C; twice for 20–25 min each at 45–50°C; and once more for 10 min at 22°C. The thoroughly washed membrane was then developed with the ECL System (ThermoFisher Scientific) using the manufacturer's protocol. Chemiluminescence from biotinylated RNA/genomic DNA was captured using a ChemiDoc™ Imaging System.

### LC-MS protocol

A Bruker maXis Impact Quadrupole Time-of-Flight LC/MS System was used for the analysis. The system consists of an Agilent 1200 HPLC and a Bruker maXis Impact Ultra-High Resolution tandem TOF (UHR-Qq-TOF) mass spectrometer. The Software used was Compass 1.5. For the MS, the ionization mode was Negative Electrospray Ionization (-ESI). Gas Temp: 180°C. Gas Flow: 8 l/min. Nebulizer: 2 bar. Capillary Voltage: 3000 V. Mass Range: 50–1500 Da. Calibrant: Sodium Formate. For the HPLC, a Spursil C18 column with 3 micron particle size, 30 mm length × 3.0 mm diameter (Dikma Technologies) was used. The column temperature was: 30°C. For the solvent gradients, Solvent A was water with 0.1% formic acid; and Solvent B was acetonitrile with 0.1% formic acid.

## RESULTS AND DISCUSSION

### Biotinylation of diverse DNA and RNA GQs

**Figure 1.**
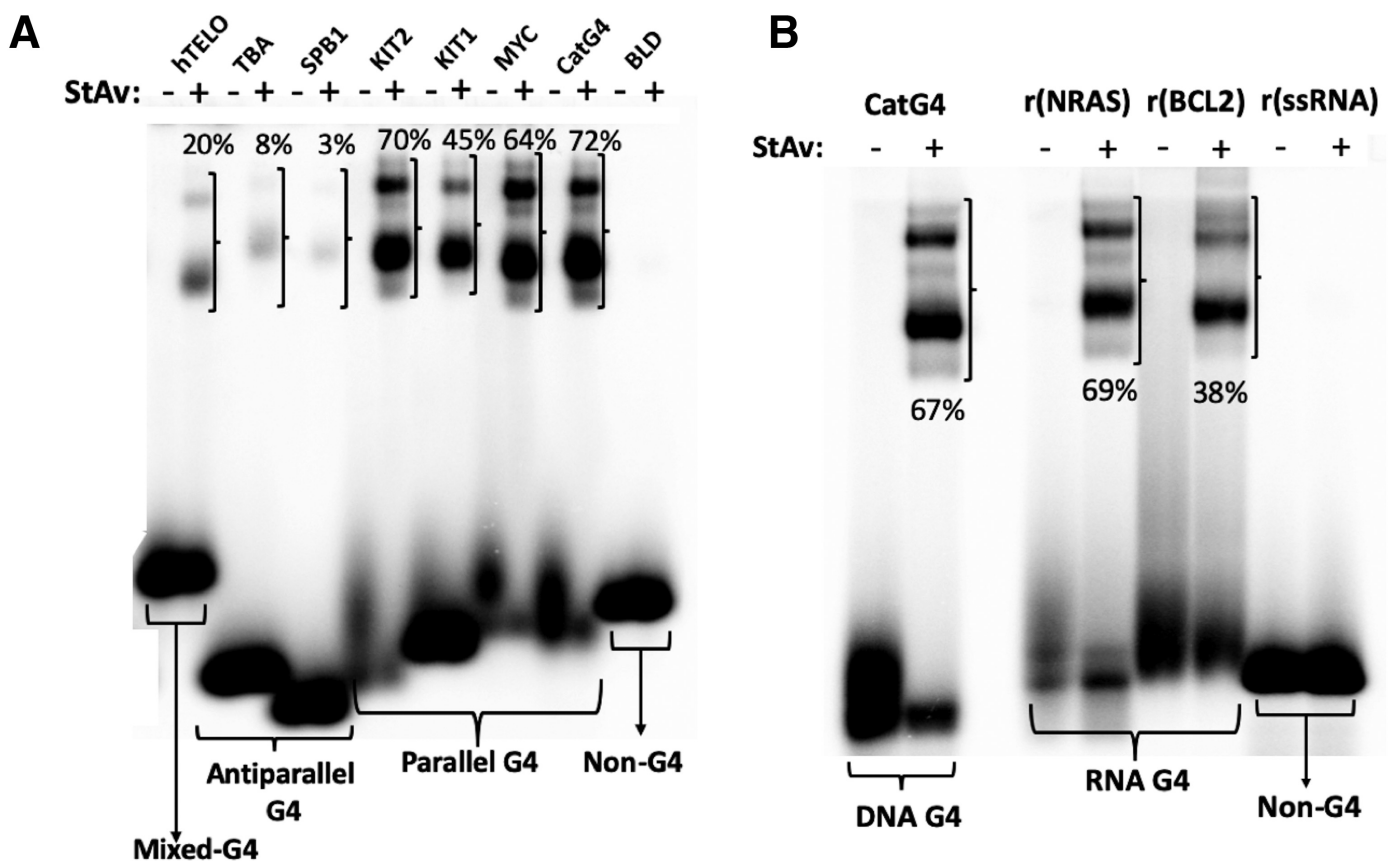
Testing of self-biotinylation by various GQ-forming DNAs (**A**) and RNAs (**B**) as well as by a DNA (‘BLD’) and an RNA (‘r(ssRNA)’) that do not fold to GQ. Both panels A and B show 7.5% non-denaturing gels run in 50 mM TBE buffer at room temperature. ‘StAv’ refers to StAv, where it has been added subsequent to the standard biotinylation reaction (marked ‘+’) or where it has not been added (marked ‘−’). The percent values shown in a given ‘+’ lane refer to the percentage of DNA in that lane that has been mobility-shifted by virtue of complexation of the biotinylated DNA with the added StAv.

As described in the Introduction, no DNA or RNA folded structure other than GQs have been shown either to bind hemin or to activate such bound hemin toward oxidative activity. GQs, however, are a highly polymorphic class of structures, with a variety of strand orientations and topologies (30). We wished, first, to investigate, whether DNA GQs of different strand orientations (all-parallel, all-antiparallel and mixed orientation (9,30)) as well as RNA GQs (which invariably form all-parallel stranded GQs (10)) could all self-biotinylate by way of the peroxidase activity of hemin complexed to them (sequences given in Table 1) (8–10). 5′-^32^P-labeled all-parallel DNA GQs (‘CatG4’, ‘MYC’, ‘KIT1’ and KIT2), all-antiparallel DNA GQs (‘SPB1’ and ‘TBA’), a mixed strand-orientation DNA GQ (‘hTELO’) as well as parallel RNA GQs (‘rNRAS’ and ‘rBCL2’) were subjected to standard biotinylation reactions (see ‘Materials and Methods’ section); together with a DNA oligonucleotide (‘BLD’) and a RNA oligonucleotide (‘r(ssRNA)), that are not capable of forming GQs (8–10). Following quenching of the biotinylation reactions, the DNAs and RNAs were treated with StAv and analyzed in non-denaturing gels. Figure 1 shows that the parallel GQs, whether DNA or RNA, were efficiently biotinylated (∼38–72%); whereas the antiparallel GQs were also biotinylated at lower but detectable levels. By contrast, no trace of biotinylation was seen with the negative control oligonucleotides, BLD and r(ssRNA).

The biotinylation of all the GQs examined here is significant for the anticipated biotinylation experiments to be carried out *in vivo*; even low levels of biotinylation (as opposed to *no* biotinylation) of the pertinent DNA or RNA sequences should enable their pulldown using StAv, followed by NextGen sequencing. Recently, indeed, RNA self-biotinylation has been characterized *in vitro* in a separate study (31).

### How many biotins covalently attach to each CatG4?

The tight and highly specific biotin-StAv interaction provides, in principle, a convenient approach for quantitating the number of biotins that covalently attach to each GQ-forming oligonucleotide under our reaction conditions. However, the use of tetrameric StAv (capable of binding up to four biotins/biotinylated DNAs) complicates such an estimation. Indeed, in a standard experiment involving GQ-forming oligonucleotides, multiple StAv-retarded gel bands of biotinylated GQ are seen (such as in Figure 1), which are difficult to interpret in terms of binding stoichiometries.

**Figure 2.**
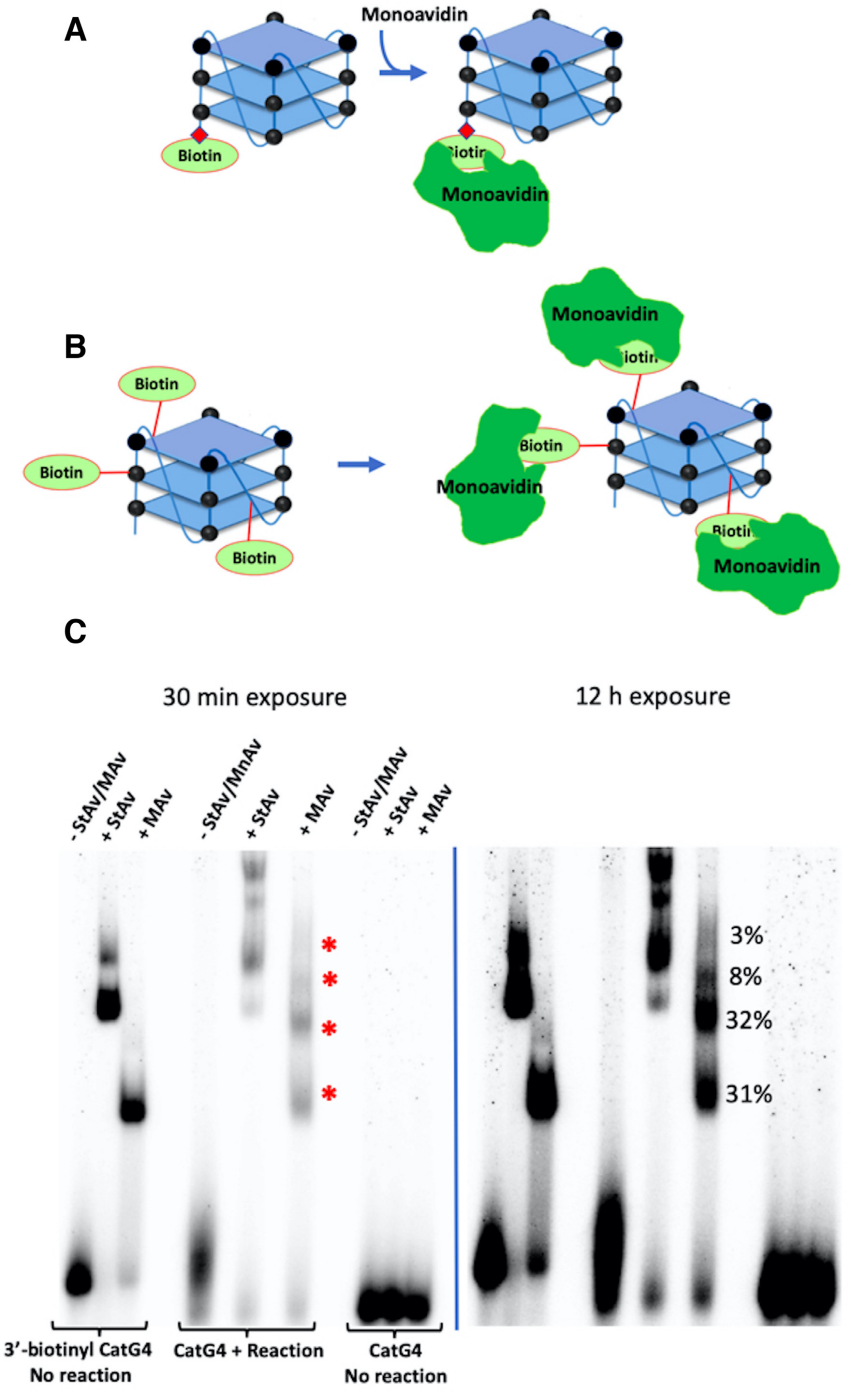
How many biotins attach to each ‘CatG4’ GQ? (**A**) Design of the reference complex between a synthetic, singly biotinylated ‘CatG4’ DNA and its 1:1 complex with monoavidin(MAv). (**B**) Expected 1:1 complexation of monoavidin with biotin moieties attached to a ‘CatG4’ DNA *via* peroxidation of a BT substrate by the ‘CatG4’s’ oxidative complex formed with hemin. (**C**) Two exposures of a native polyacrylamide gel showing the appearance of up to four monoavidin-retarded bands (shown with red asterisks) formed from the reaction described in (B).

To address this problem, we decided to use an MAv (SAvPhire Monomeric StAv or ‘monoavidin’), with its property of forming an exclusively 1:1 complex with biotin. To generate a reference complex consisting of the GQ forming ‘CatG4’ DNA oligonucleotide with a single appended biotin, in turn complexed to a single monoavidin molecule, we used a ‘CatG4’ oligonucleotide chemically synthesized with a 3′-appended biotin moiety (‘3′-biotinyl CatG4’). Figure 2A schematically shows such a DNA and its expected 1:1 complex with monoavidin. Figure 2B shows the expected binding scenario of a multiply biotinylated ‘CatG4’ with monoavidins.

Figure 2C shows that while 3′-biotinyl ‘CatG4’ mixed with StAv generates two retarded bands of uncertain DNA:protein stoichiometry (‘3′-biotinyl DNA; no reaction; + StAv’), when it is mixed instead with monoavidin (‘3′-biotinyl DNA; no reaction; + MAv’), a single, clearly defined, monoavidin-retarded band results. This result provides us with a positive control for investigating the extent of biotinylation of ‘CatG4’ under our reaction conditions, based on the presumption that each biotin covalently linked to a given CatG4 molecule would bind a single monoavidin (shown conceptually in Figure 2B). Figure 2C shows two different exposures of the same native gel. Two strong retarded bands (corresponding to two biotins appended to one CatG4) can be seen but up to a total of four retarded bands can be discerned (shown by red asterisks in the lane marked ‘CatG4 + reaction + MAv’). The percentages of these retarded bands are given in Figure 2C. In summary, with folded ‘CatG4’ (a 21-nt, three G-quartet GQ), 2–4 biotins typically attach under our reaction conditions.

We asked the following further questions: (i) how reproducible are the levels of biotinylation observed for individual GQs under a given reaction condition (such as shown in Figure 1)? Furthermore, (ii) is there a higher efficiency of biotinylation with a higher (50 μM rather than 5 μM) heme concentration? Supplementary Figure S1a shows a gel reporting the standard biotinylation of GQs with 5 μM heme. Two independent reactions were carried out, and the numbers shown in the gel (panel a) reflect that. The percentage values of monoavidin- versus StAv-retarded DNA for both the TBA (antiparallel) and the MYC (parallel) GQs are mutually consistent. Further, the error bars (representing deviations from the mean) are small, showing a high reproducibility of the biotinylation reaction under a given reaction condition. Supplementary Figure S1b shows that notable biotinylation enhancements (97% for MYC and 15% for TBA) are achieved with 50 μM as opposed to 5 μM heme. Again, the reproducibility of biotinylation levels seen with two independent experiments using 50 μM heme is high.

### Biotinylation competition experiments with large excesses of ssDNA or dsDNA in dilute and concentrated (gelated) solutions

Of the variety of secondary and tertiary structures formed by natural RNA and DNA, only GQs have been shown to bind hemin. Hemin neither binds to ssDNA or dsDNA nor is it activated toward oxidative catalysis by those DNA folds (21,24). If self-biotinylation is intended to be a reliable method for tagging GQs *in vivo*, it would be necessary to estimate the degree to which, in complex mixtures of ss/dsDNA and GQs, promiscuous labeling of the ds or ss DNA might occur.

We investigated this first in dilute DNA solution, with 10 nM ‘CatG4-ext’ DNA co-dissolved with very large (10^4^-fold) excesses of either ‘ssDNA’ or ‘dsDNA’. This particular molar ratio was chosen based on rough calculations on the ratio of the size of the human genome (∼ 3 × 10^9^ bp) to the reported number of GQ-capable sites within the genome (7 × 10^5^ bp). (32).

**Figure 3.**
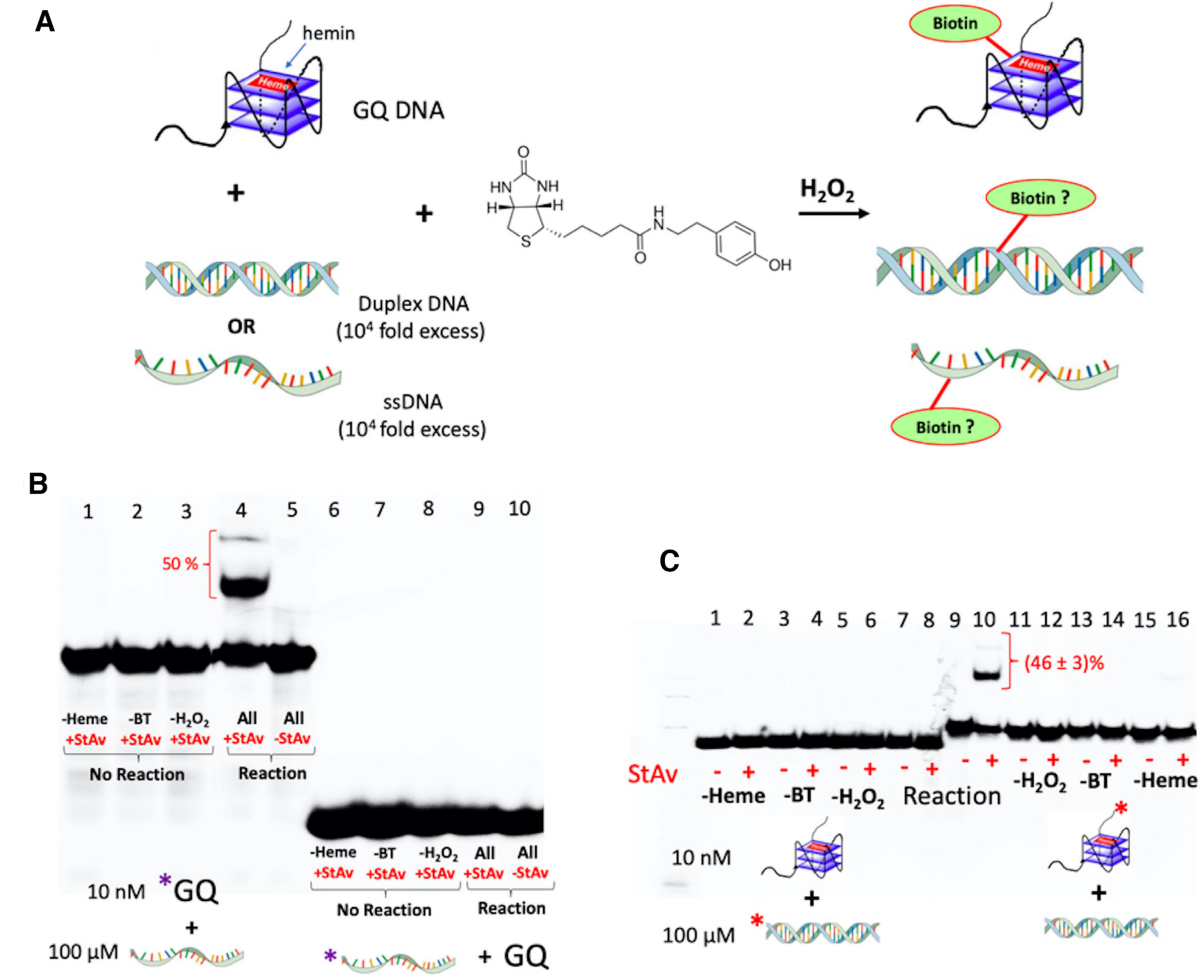
The specificity of GQ biotinylation over ss or dsDNA in dilute co-solutions. (**A**) A schematic diagram for the experiment, in which co-dissolved 10^4^:1 molar ratios of ‘ssDNA’:‘CatG4-ext’ or of ‘dsDNA’:‘CatG4-ext’ are treated with hemin, H_2_O_2_ and biotin-tyramide (BT). (**B** and **C**) Native gels showing reciprocally ^32^P-end-labeled ‘CatG4-ext’ (10 nM) co-dissolved 100 μM ‘ssDNA’ (B) or 100 μM ‘dsDNA’ (C). Upon treatment with StAv, retarded mobility (biotinylated) bands (indicated by red brackets) were observed and then quantitated relative to the unbiotinylated DNA in those same lanes.

Figure 3A illustrates the concept of the experiment. Figure 3B shows the results of ‘CatG4-ext’ co-dissolved (10 nM) with ‘ssDNA’ (100 μM), the two DNAs being reciprocally ^32^P-labeled. The DNA mixture was treated with hemin, BT and H_2_O_2_, purified by ethanol purification, mixed with StAv, and run in a native polyacrylamide gel. Figure 3B shows that StAv-shifted gel bands (∼50% of the total DNA in that lane, indicated with a red bracket) are visible only in the lane containing a mixture of ^32^P-labeled ‘CatG4-ext’ DNA co-dissolved with unlabeled ‘ssDNA’; and, that no trace of such shifted bands can be seen from the lane containing ^32^P-labeled ‘ssDNA’ mixed with unlabeled ‘CatG4-ext’ DNA. The biotinylation of ‘CatG4-ext’ is therefore completely specific, even in the background of a 10^4^-fold excess of ‘ssDNA’. Figure 3C shows results almost indistinguishable from those seen in Figure 3B, except, in this experiment, 10^4^-fold excess ‘dsDNA’ replaces 10^4^-fold excess ‘ssDNA’.

### Does GQ biotinylation occur even in a highly concentrated DNA solution?

**Figure 4.**
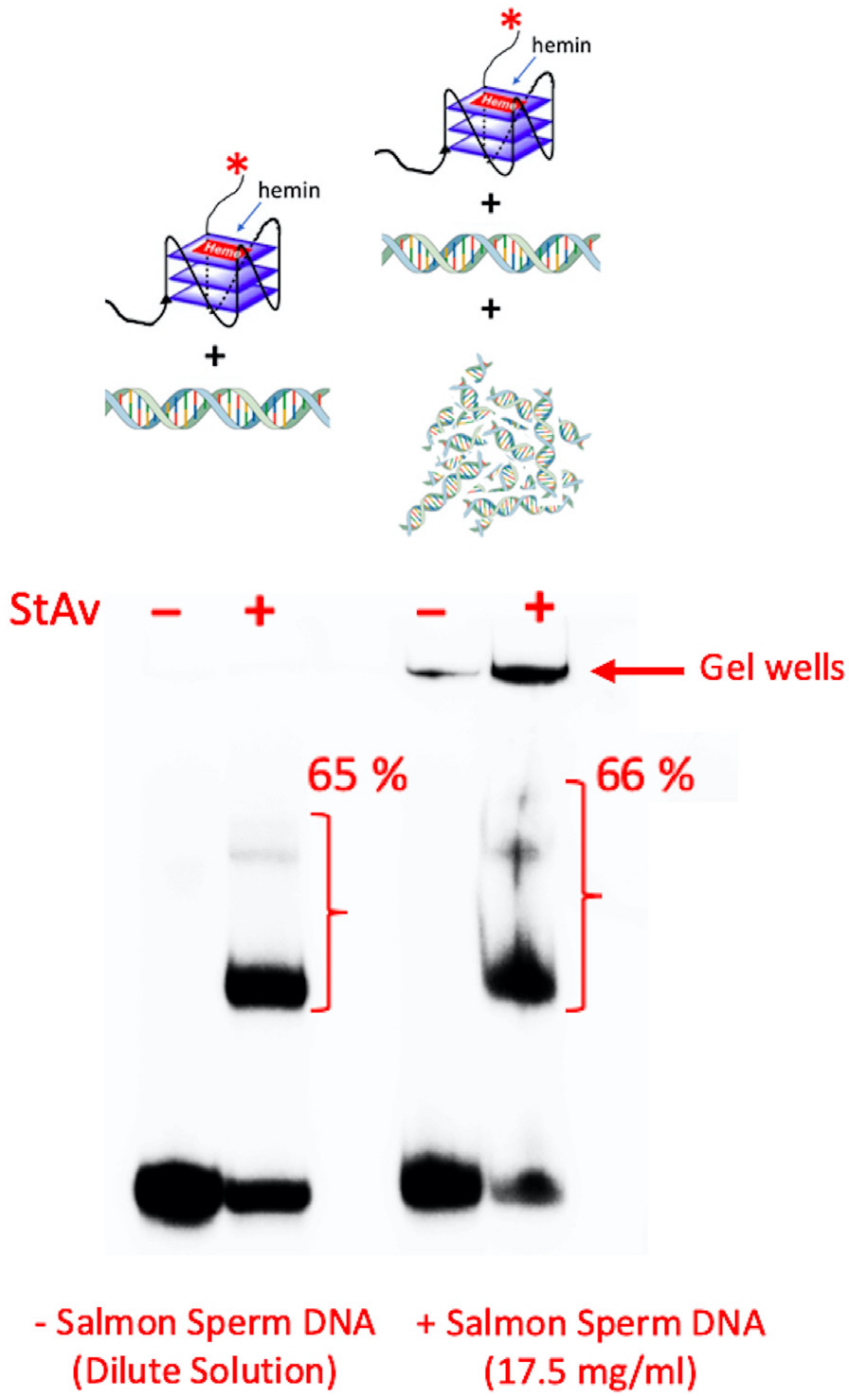
The specificity of GQ biotinylation over dsDNA in highly concentrated (gelated) dsDNA solutions. Co-dissolved 10^4^:1 molar mixtures of ‘dsDNA’:‘CatG4-ext’ were treated with hemin, H_2_O_2_ and biotin-tyramide (BT) either in dilute solution (‘-Salmon Sperm DNA’) or in a highly concentrated dsDNA solution (‘Salmon Sperm DNA: 17.5 mg/ml’)

What happens when the biotinylation experiment, as above, is carried out not in dilute solution but in a milieu of highly concentrated, gelated DNA, such as might be found in a cellular nucleus? To explore this question, we performed a new competition experiment using ^32^P-labeled 10 nM ‘CatG4-ext’ and 100 μM ‘dsDNA’, either with no further added DNA (dilute solution) or within a highly concentrated (gelated) DNA environment containing a very high concentration of sheared salmon sperm genomic DNA (17.5 mg/ml), in QD Buffer. Under these DNA and salt conditions, such a solution has been shown rigorously to form a viscous and isotropic gel (33); this was also our own observation. Figure 4 shows that under these two very different solution conditions, biotinylation of the GQ formed by the ‘CatG4-ext’ remains comparably efficient in the dilute solution as well as in the highly concentrated DNA solution.

It could be argued that within the gelated DNA regime generated by the salmon sperm DNA (which itself is not radiolabeled in our experiment) some promiscuous labeling of the concentrated salmon sperm DNA may occur. To address this issue, we describe experiments, below, to determine what the ‘active zone’ (or spatial restriction) for biotinylation may be around a GQ-complexed hemin that oxidizes BT to its phenolic radical, in turn capable of labeling DNA.

### What is the spatial restriction of biotinylation in a GQ-duplex chimeric DNA?

**Figure 5.**
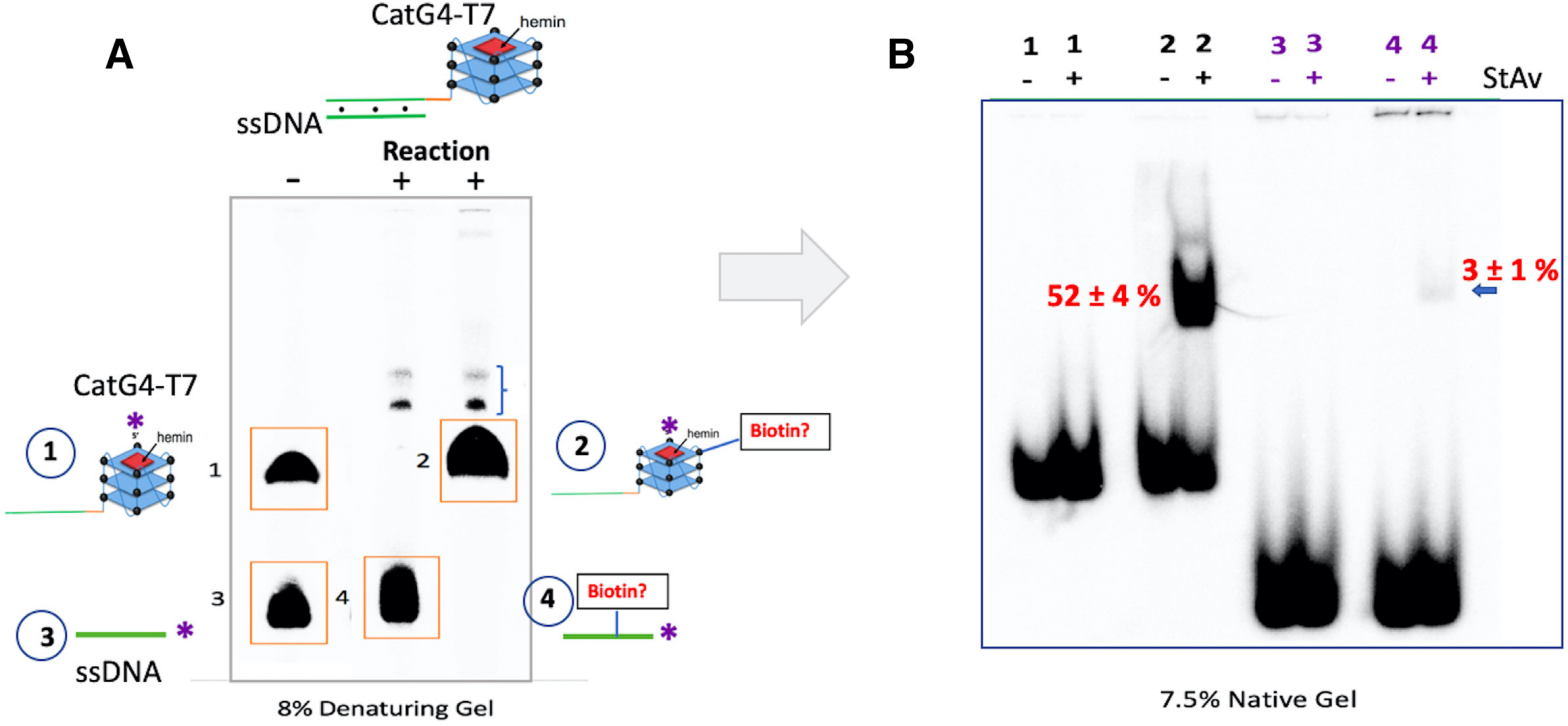
The distance range of biotinylation. (**A**) Top, the design of a duplex-GQ chimera, 46-nt ‘CatG4-T7’ hybridized to 22-nt ‘ssDNA’. Bottom, an 8% denaturing gel showing the individually ^32^P-labeled component oligonucleotides, ‘CatG4-T7’ (band ‘1’) and ‘ssDNA’ (band ‘3’) that make up the duplex-GQ chimera. Bands ‘2’ and ‘4’ represent show, respectively, post-biotinylation ^32^P-labeled ‘CatG4-T7’ (out of the duplex-GQ chimera containing non-radiolabeled ‘ssDNA’) and post-biotinylation ^32^P-labeled ‘ssDNA’ (out of the duplex-GQ chimera containing non-radiolabeled ‘CatG4-T7’). The minor bands shown with a bracket (}) represent inter-strand crosslinked minor products formed between ‘ssDNA’ and ‘CatG4-T7’. (**B**) A native gel showing purified DNA isolated and purified from bands 1–4 shown in the denaturing gel (in A), run with either StAv added (+) or not added (−). The numbers shown in red indicate the percentage of total DNA StAv-shifted (and are therefore biotinylated) in the relevant lanes.

To determine if non-GQ DNA duplex elements physically linked and therefore highly proximal to a GQ (such as might be found within a living cell) might also be targets for biotinylation, and to determine how far away from the CatG4-bound hemin such biotinylation might occur, we hybridized together the 46-nt ‘CatG4-T7’ and 22-nt ‘ssDNA’ to generate a GQ-duplex chimera consisting of a 22-bp duplex pendant from the ‘CatG4’ GQ by an AAA nucleotide linker (shown schematically in Figure 5A). Biotinylation was performed with reciprocally 5′-^32^P-labeled ‘CatG4-T7’ and 5′-^32^P-labeled 22-nt ‘ssDNA’ (bound, to unlabeled ‘ssDNA’ and ‘CatG4-T7’, respectively), and complexed with hemin.

After the biotinylation reaction, a denaturing gel (Figure 5A) was used, first, to separate and purify the radiolabeled ‘ssDNA’ and ‘CatG4-T7’ strands from their unlabeled, partially complementary strands. The purified DNA was then mixed with StAv and analyzed in a native gel (Figure 5B) to quantitate the proportion of the purified, ^32^P-labeled oligonucleotide that had undergone biotinylation. Figure 5B shows that ∼52% of the radiolabeled ‘CatG4-T7’ strand was StAv-shifted under our reaction conditions. Of course, this longer of the two oligonucleotides participates in both the GQ and duplex domains of the duplex-GQ chimera. The radiolabeled ‘ssDNA’, however, participates only in the duplex portion of the chimera, and Figure 5B shows that only ∼3% of this oligonucleotide is StAv-shifted (Supplementary Figure S2 shows a longer exposure of this gel).

**Figure 6.**
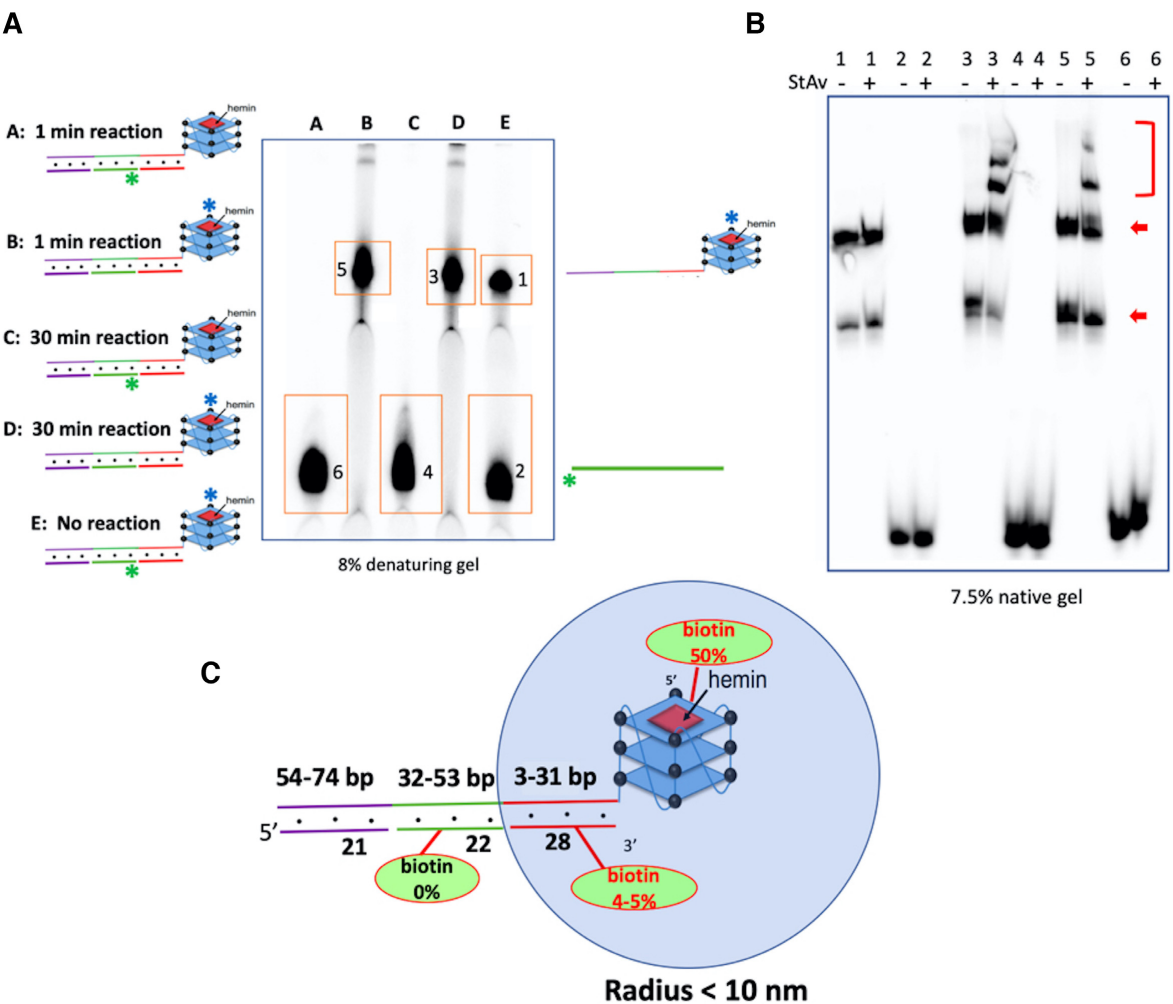
Examination of biotinylation in an extended duplex linked to a GQ. (**A**) Does biotinylation extend past 31 bp in a duplex linked to a GQ? To the left are shown a GQ-duplex chimera consisting of three short oligonucleotides, ‘Comp-1’, ‘ssDNA’ and ‘Comp-3’, hybridized simultaneously to different stretches of the tailed GQ-forming oligonucleotide ‘CatG4-ext2’. Here, only either ‘CatG4-ext2’ or ‘ssDNA’ were 5′ ^32^P-labeled (shown, respectively, as a blue asterisk and a green asterisk). The denaturing gel shows the radiolabeled ‘CatG4-ext2’ or ‘ssDNA’ from the complete GQ-duplex chimera, either biotinylated under the specified conditions or not. (**B**) Native gel showing StAv-shifted bands from DNA species 1–6 following purification from the denaturing gel shown in A. The bands shown with the red bracket are the StAv shifted bands. The multiple bands seen from ‘CatG4-ext2’ (shown with red arrows) represent different folded conformers formed in the native gel by this large oligomer. (**C**) The effective radius of biotinylation (∼10 nm, representing a duplex of ∼31 bp), estimated from the above experiments.

To test the further reaches of a longer duplex linked to a GQ, we generated a different chimeric DNA complex (shown in Figure 6A), in which the duplex component is 74 bp long. As shown in this figure, three short oligonucleotides ‘Comp-1’, ‘ssDNA’ and ‘Comp-3’ (with only ssDNA being 5′-^32^P-labeled) were hybridized simultaneously to different stretches of the tailed GQ-forming oligonucleotide ‘CatG4-ext2’, to generate a quasi-continuous duplex appended to the GQ. The denaturing gel (Figure 6A) shows the dissociated, ^32^P-labeled ‘CatG4-ext2’ and ‘ssDNA’ out of the complete GQ-duplex chimera, following either biotinylation under the specified conditions or not biotinylation. Figure 6B shows a native gel that highlights StAv-shifted bands (shown with a red bracket) obtained from the DNA species 1–6 shown in Figure 6A following their purification and mixing with StAv. Crucially, it can be seen that *no* StAv-shifted band is seen from bands 4 and 6 (representing ‘ssDNA’). Thus, ‘ssDNA’, hybridized 32–53 bp away from the GQ in this construct, is not biotinylated at all.

Figure 6C summarizes the above data and proposes that the effective radius of biotinylation estimated from the above experiments is ∼10 nm (representing a duplex of ∼31 bp) away from the GQ.

### Covalently appended biotins are distributed along the entire length of a GQ

**Figure 7.**
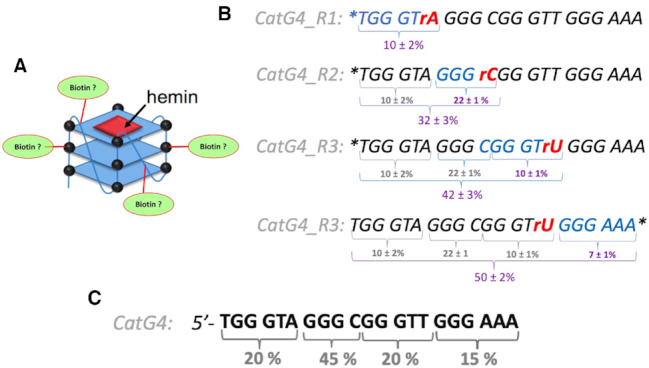
The distribution of covalently appended biotins along the length of CatG4 DNA. (**A**) A schematic of the question being posed, about the distribution of biotin along the length of the CatG4 oligonucleotide. (**B**) The sequences of the four ‘CatG4’ variants, CatG4_Rx (where *x* = 1–3). The nucleotide marked in red is the single ribonucleotide within each of these oligonucleotides. The asterisk shows the site of ^32^P-labeling (at the 5′ or the 3′ end). The numbers shown below each sequence indicate raw percentages of StAv-shifted bands relative to total DNA in a given gel band (such as shown in Supplementary Figure S2). The numbers obtained were from two independent sets of measurements. (**C**) Absolute percentages of the likelihood of biotin localization along segments of the total sequence of the ‘CatG4’ oligonucleotide.

We were interested to determine the distribution of biotin attachment along the 21-nt length of the ‘CatG4’ oligonucleotide (shown schematically in Figure 7A). Does biotinylation occur, for instance, uniformly along the length of ‘CatG4’, or preferentially to its 3′ or 5′ ends? To address this question, we devise a number of variants of ‘CatG4’, all of which had the same base sequence as CatG4 itself but incorporated a single ribonucleotide at different locations within the deoxyribonucleotide.

Figure 7 shows the sequences of three CatG4 variants, ‘CatG4_R1’, ‘CatG4_R2’ and ‘CatG4_R3’. The nucleotide marked in red in each case is the single embedded ribonucleotide (Figure 7B). Asterisks show the site of ^32^P-labeling (i.e. either at the 5′ or the 3′ end) in a given oligonucleotide. Following biotinylation under the standard conditions described above, the 5′- or 3′-labeled ‘CatG4_R1’ to ‘CatG4_R3’ oligonucleotides were treated, first, with hot alkali, to cleave their phosphodiester chains at the embedded ribonucleotides. Second, the two resulting pieces obtained from each of the oligonucleotides were separated and purified by denaturing gel electrophoresis. Third, the extent of gross biotinylation (i.e. the coupling of at least one biotin to a given ^32^P-end-labeled DNA fragment, relative to that in the ^32^P-end-labeled but uncleaved ‘CatG4’) was determined by StAv band-shift analysis. Supplementary Figure S3 shows a schematic for this experimental approach, and Supplementary Figure S4 shows the overall experimental data obtained from an analysis of the oligonucleotide ‘CatG4_R1’. Quantitation of the DNA bands (such as shown in Supplementary Figure S4C) is displayed in Figure 7B. The numbers shown below each sequence of ‘CatG4_Rx’ (where *x* = 1–3) indicate raw percentages of StAv-shifted bands relative to the total DNA in a given gel band. Figure 7C tabulates the absolute percentages of biotin localization along the full length of the ‘CatG4’ oligonucleotide. Our approach does not attempt to identify individual bases or nucleotides or sites within them as specific atomic loci for biotin attachment; nevertheless, it can be seen that biotins are more or less evenly distributed along the length of ‘CatG4’. The stretch …GGGC… within ‘CatG4’ has somewhat higher levels of biotinylation relative to the other three quadrants; the reason for this is not immediately clear. It is conceivable that the single dC in this stretch or its neighbouring phosphates are preferred sites for biotin attachment.

To obtain a level of deeper insight into the sites of GQ biotinylation, we carried out chemical and mass spectrometric experiments on biotinylated DNA (and RNA) GQs. First, we attempted to degrade biotinylated and unbiotinylated DNA (CatG4) GQs with the highly efficient Nuclease P1, to mononucleotides (5′-NMPs), such that the biotinylated mononucleotides could be analyzed by mass-spectrometry. However, we found that while the unmodified GQ was readily degraded by P1, biotinylated GQ was poorly and incompletely degraded. Attempting an alternative strategy, we tested whether the GQ biotinyl labels were hot base-labile (heating to 90° at neutral pH does *not* destroy the GQ biotinyl labels—ref. 29). Accordingly, the DNA (CatG4) GQ was biotinylated in a standard reaction, and a portion of the resulting DNA was subjected to heating at 90°C in 0.1 M NaOH. Supplementary Figure S5A shows that upon such treatment, the level of DNA biotinylation (indicated by the proportion of DNA band-shifted by StAv binding in a native gel) decreased from 58% to only 8% of the DNA. Interestingly, ∼9% of the starting DNA broke down into smaller fragments (such as might be expected if alkylation/biotinylation had occurred to the N7 position of guanines, for example).

The above data suggested that the *phosphate* groups of the GQ were important (though not necessarily exclusive) sites for biotinylation—DNA phosphotriesters are known to be notably base-labile (34). To test whether BT or a derivative was indeed being released from base-treated GQs (both RNA and DNA), a mass-spectrometry based quantitation was carried out (experimental scheme shown in Supplementary Figure S5B). Precisely equal amounts of a biotinylated and, separately, unbiotinylated RNA GQ (NRAS) and also biotinylated and unbiotinylated DNA GQ (CatG4) were purified first, in parallel, by two successive ethanol precipitations each, then hot base-treated, followed by analysis and quantitation of their contents by LC-ESI-MS.

Supplementary Figure S6 shows, first, that in the hot-based treated RNA solutions, the two most abundant base-hydrolyzed NMP products, GMP (‘rG’ in Supplementary Figure S6A and B) and AMP (‘rA’) were detected in equivalent quantities in the biotinylated and unbiotinylated samples, thus serving as internal standards for quantitation of any released biotin-tyramide in these same solutions. Indeed, an at least *4-fold* excess of BT was measured in the base-treated biotinylated RNA solution (relative to the unbiotinylated RNA). The DNA analysis (Supplementary Figure S6C and D) showed an absence of hydrolyzed dNMP products, and found an at least two-fold excess of BT in the hot-base treated biotinylated sample, relative to the unbiotinylated sample.

### Do GQ-binding ligands compete with heme with respect to GQ self-biotinylation?

**Figure 8.**
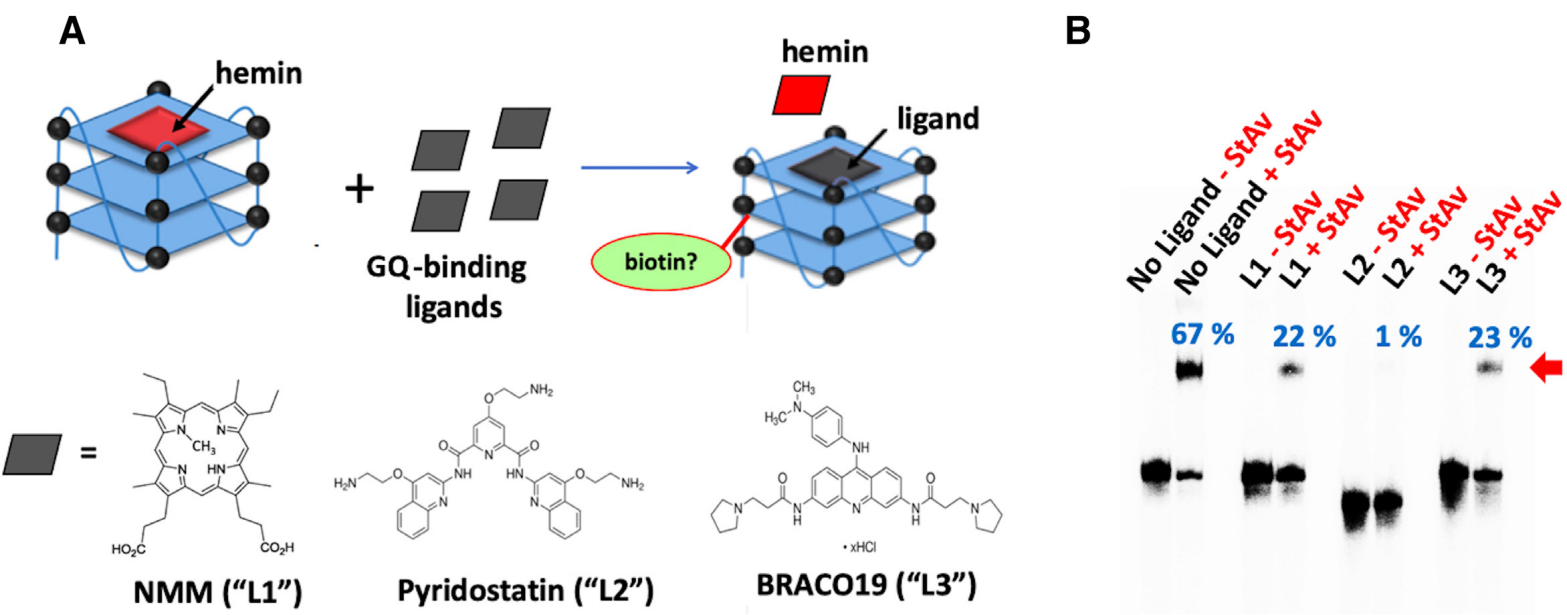
The competitive effect of GQ-binding ligands. (**A**) A schematic showing of 10 min treatments of ∼1 μM heme–GQ complex (made by complexation of 1 μM ‘CatG4-ext’ with 5 μM hemin) with 20 μM of, individually, NMM, pyridostatin and BRACO19 GQ-binding ligands. (**B**) A native gel showing StAv-retarded bands of biotinylated CatG4-ext complexed with added StAv (shown with a red arrow). The percentage of this retarded complex (with respect to the total DNA in a given lane) is indicated in blue above each lane.

As discussed above, a number of tight-binding, synthetic GQ ligands have been reported in the literature. These include N-methylmesoporphyrin IX (NMM; ‘L1’ in Figure 8A) (16), pyridostatin (‘L2’ in Figure 8A) (35); and BRACO19 (‘L3’ in Figure 8A) (36), all of which bind to GQs by end-stacking upon terminal G-quartets, much as hemin does (37). It might therefore be expected that addition of excess GQ-ligands, that compete for binding with hemin, should reduce GQ–hemin concentration and hence, the overall levels of GQ self-biotinylation. Figure 8B shows that this is indeed the case. Individual competition with four-fold excesses (over hemin) of the three ligands leads to significant decreases in the overall percentage of StAv-shifted gel bands (67% overall biotinylation seen in the absence of any competing ligand; 22–23% in the case of competition with NMM or BRACO19, and only 1% in the presence of pyridostatin). To get more precise and quantitative data on these inhibitions of hemin-mediated biotinylation by GQ binders, levels of ‘CatG4-ext’ biotinylation were tested in the presence of 5 μM hemin and varying concentrations of the three GQ ligands. These data are shown in Supplementary Figure S7. It can be seen that ∼100 μM concentrations of either NMM or BRACO19 are required to fully abolish ‘CatG4-ext’ biotinylation; whereas, only ∼10 μM Pyridostatin is required to do the same. The reported binding affinities of the three ligands to GQs are roughly in the same range as that of hemin (16,35–37). The data shown here, however, suggest a superior binding affinity of Pyridostatin, relative to the other two ligands, to the specific GQ formed by ‘CatG4-ext’.

Importantly, the above data confirm, again, that it is GQ-bound hemin (as opposed to free-floating hemin) that is responsible for the observed GQ self-biotinylation. This result also builds confidence that when GQ self-biotinylation is sought for *in vivo* using this method, the identification of purely GQ DNA/RNA-generated self-biotinylation (as opposed to adventitious biotinylation caused by non-GQ peroxidases or other oxidases) can be confirmed by checking the competitive impact of GQ-ligands on intracellular DNA/RNA biotinylation. This is because these three chemically diverse GQ ligands are uniquely directed to binding GQs and not the active sites of proteinaceous oxidases within living cells.

### Evidence for GQ-mediated self-biotinylation for tagging RNA and DNA in living tissue

**Figure 9.**
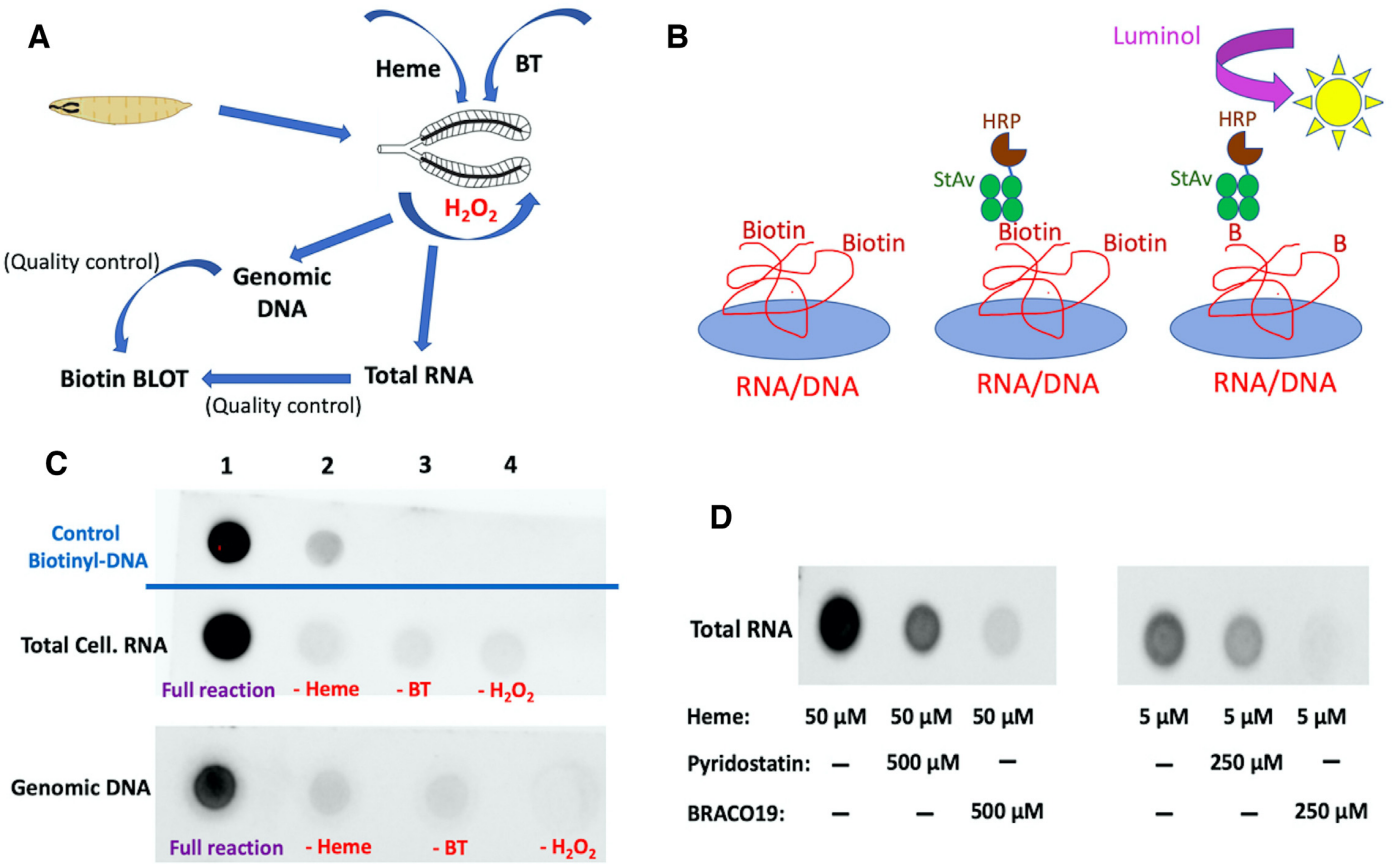
Biotinylation of RNA and DNA within live *Drosophila melanogaster* salivary glands. (**A**) A schematic diagram showing the design of the experiment. (**B**) Design of biotin dot-blots of total cellular RNA and genomic DNA. (**C**) Developed biotin dot blots from 2–5 μl (DNA = 200 ng; RNA = 400 ng for all dot blots) of total gland RNAs and genomic DNA extracted from live *Drosophila* larvae that have been treated with 50 μM heme, 3 mM BT and pulsed briefly (2–3 min) with 10 mM H_2_O_2_ followed by quenching. Blots ‘1’ show chemiluminescence from the full reaction, where all the above reagents are present; ‘2’, ‘3’ and ‘4’ show negative controls, where one of the participating reagents at a time is left out. The positive control spots, ‘Control Biotinyl-DNA’ show a standard 3′-biotinylated DNA spotted at two different concentrations: 2 ng (Spot 1) and 0.2 ng (Spot 2). (**D**) Developed biotin dot blots from 2–5 μl (DNA = 200 ng; RNA = 400 ng for all dot blots) of total cellular RNAs isolated from live salivary glands that have been treated with 50 μM heme plus 500 μM GQ-ligand (or 5 μM heme plus 250 μM GQ-ligand) and 3 mM BT, all pulsed briefly (2–3 min) with 10 mM H_2_O_2_ followed by quenching.

Clearly, GQ self-biotinylation can work efficiently *in vitro*. However, its application *in vivo* could, in principle, encounter unanticipated challenges including, potentially, a too-low oxidative activity of GQ–hemin in the intracellular milieu. We elected to try a few key experiments to test GQ–hemin peroxidase activity in living tissue. Initially, we chose to investigate salivary glands dissected from the larvae of *D. melanogaster* (Figure 9A). The goal of these experiments was to test for detectable levels of biotinylation within total tissue RNA and genomic DNA extracted from the glands following treatment of the glands with hemin, BT and H_2_O_2_. It was also important to provide evidence that any observed biotinylation was not adventitious but a likely consequence of intracellular heme–GQ oxidative activity.

The feeding of low concentrations of heme to mammalian cells in tissue culture has been successfully exploited by Ting and coworkers to optimize the activity of heme-based recombinant peroxidases that they have expressed in such cells (38,39). We used the concentrations of reagents used by those studies as starting points but adjusted them to suit our experimental system. To assay for covalent biotinylation within total RNA as well as genomic DNA extracted from treated whole salivary glands, a ‘biotin blot’ protocol was devised (Figure 9B), whereby rigorously purified RNA, and separately, DNA from hemin, BT and H_2_O_2_-incubated salivary glands (as well as from negative control experiments in which incubation with one key reagent at a time, hemin, BT or H_2_O_2_, was omitted) were immobilized *via* UV crosslinking to a nylon membrane, and then probed for biotinylation status by the specific binding of commercially available StAv-HRP conjugates and subsequent oxidized luminol chemiluminescence (Figure 9B). As a positive control for this protocol, we used pure 3′-biotinyl CatG4 (Figure 1; *vide infra*) in two different amounts (2 and 0.2 ng), which were spotted directly onto the membrane and crosslinked.

Salivary glands were dissected from live third instar *Drosophila* larvae using standard methods into phosphate-buffered saline (PBS) buffer, and were incubated with 5–50 μM heme and 3 mM BT. They were then briefly (2–3 min) pulsed with 10 mM H_2_O_2_. Total RNA and, separately, genomic DNA, were purified using commercial purification kits and 400 ng (for RNA) and 200 ng (for DNA) were spotted onto a nylon membrane using a dot blot device, followed by UV-crosslinking. How do we know that the commercial kits used in this study indeed purified genomic DNA and RNA, respectively? And, that these preparations were not contaminated by proteins from the live tissue? Three kinds of evidence are shown in Supplementary Figures S8 and 9. First, Supplementary Figure S8A, shows a 1% agarose gel running the purified total RNA aliquot and a purified genomic DNA aliquot. It can be seen that the two aliquots are indeed different from each other. Supplementary Figure S8B shows the UV-vis spectra of the purified genomic DNA and total RNA, respectively. The spectra report A_260_/A_280_ values > 1.8, consonant with nucleic acid preparations uncontaminated by proteins. Supplementary Figure S9 shows that the chemiluminescence ‘output’ signals from both 3′-biotinyl ‘CatG4’ (a positive control) and genomic DNA biotinylated *in vivo* were comparably abolished by digestion of these DNAs with active DNAase I, and were correspondingly *not* abolished by digestion of these DNAs with inactivated DNAase I.

Following the spotting of DNA/RNA upon the Hybond Nylon+ membranes, above, the membranes were blocked, washed and treated with HRP-StAv; and, finally, after extensive washing, they were developed using bound HRP-mediated luminol oxidation and the recording of chemiluminescence.

Figure 9C and D show the results. Figure 9C shows that both total salivary gland RNA and genomic DNA show evidence of strong biotinylation following treatment with hemin and BT, followed by a pulse of H_2_O_2_, whereas omission of any one of these key reagents leads to a full loss of signal. At this point, however, these results do not guarantee that the observed biotinylation of salivary gland RNA and DNA results from oxidation of BT by intracellular GQs complexed with hemin; some other kind of adventitious oxidation of BT and concomitant DNA/RNA biotinylation could, in principle, be involved. To eliminate such a possibility, we investigated biotinylation of whole intracellular RNA in the presence not only of heme but of either one of the two strong GQ-ligands, Pyridostatin and BRACO19. Figure 9D shows that co-treatment (along with hemin) of either of these compounds leads to significant (with Pyridostatin) or almost complete (with BRACO19) loss of biotinylation signal. These results broadly mirror the data obtained *in vitro* (shown in Figure 8B), though there are differences. Supplementary Figure S10 shows quantitation of the *in vivo* biotinylation suppression data. It is interesting that BRACO19 displays somewhat superior activity to Pyridostatin *in vivo* (*in vivo*, an ensemble of different GQs, each with its own binding preference to these ligands, is likely encountered); whereas, the opposite is true *in vitro*, where a *specific* GQ has been used (Figure 8). Therefore, the difference in our *in vitro* and *in vivo* results may reflect the use of a single, *defined* GQ *in vitro*, whereas the *in vivo* data represent the cumulative activity of many different GQs found within living *Drosophila* cells.

As mentioned above, these non-heme GQ ligands (structurally distinct from hemin as well as from each other) are expected *uniquely* to compete with heme for binding GQs, and not to have other inhibitory interactions with proteinaceous oxidases or peroxidases within living cells (even if one of these mutually structurally distinct ligands did, it is very unlikely that *both* would have the same effect). Furthermore, it is highly unlikely that these ligands are inhibiting the BT oxidation by comprehensively competing (at 250 μM concentration) with the outstanding phenolic substrate BT (at 3 mM) as *substrates* for oxidation by hemin–GQ or, indeed, other oxidases in the cell.

The experiments shown in Figure 9 report semi-quantitative *in vivo* data; they do not yet supply direct sequence information on the *Drosophila* RNAs and DNA sequences biotinylated by the procedure. Such an analysis is, indeed, the subject of the next phase of our investigation. Nevertheless, the data presented in Figure 9 provide compelling evidence that under the right experimental conditions, GQ–hemin-mediated nucleic acid self-biotinylation *does* work *in vivo*. As described earlier, there is now evidence of the natural sequestration of intracellular hemin by cellular RNA/DNA GQs (27); the biotin blots reported here are not sensitive enough to detect that. However, once actual sequence information is obtained on cellular RNAs and DNAs that are capable of self-biotinylation, we anticipate that the intracellular nucleic acids that naturally sequester heme will readily be identified.

## CONCLUSION

We have shown that *in vitro*, GQ self-biotinylation is (i) overwhelmingly specific for GQ over duplex or single-strands, even under conditions of very high, gelated, concentrations of DNA; (ii) distributed relatively evenly over the length of a DNA that folds to a GQ, with two–four biotinylation events occurring per 21-nt ‘CatG4’ oligonucleotide under our reaction conditions; (iii) that the effective range of biotinylation is <10 nm from the GQ itself. (iv) We carried out a preliminary investigation of whether the biotinylation protocol could be applied to live tissue and found compelling evidence that intracellular nucleic acid biotinylation did occur, as indicated by a biotin-blot procedure. (v) At last, we used GQ-specific ligands, that compete for GQ binding with hemin, to show that application of such ligands in competition with hemin lowered or abolished self-biotinylation levels, both *in vitro* and *in vivo*.

On the cumulative strength of these above data, we believe that the self-biotinylation procedure can indeed be productively used to tag, identify and pull down DNAs and RNA folded into GQs within living cells. We anticipate that at the level of ChIP-Seq and subsequent Next-Gen DNA Sequencing, GQs that are naturally interacting with heme within the cell (such as demonstrated in ref. 27) will be identified without the provision/feeding of extraneous heme to the cells/tissues being examined. However, the feeding of extraneous heme will likely help to identify additional DNA and RNA sequences that show a *capability* to form GQs within the cell, perhaps in response to specific cellular conditions or environmental stimuli. Where fed extraneously, heme is expected to functionally resemble GQ-stabilizing ligands, such as NMM, BRACO19, pyridostatin and others, in terms of their strengths and limitations for use in probing for GQs within cells.

## Supplementary Material

gkaa281_Supplemental_File
