# Supplementary material for: High specificity and tight spatial restriction of self-biotinylation by DNA and RNA G-Quadruplexes complexed in vitro and in vivo with Heme
Source: Nucleic Acids Res. 2020 Apr 24;48(10):5254–67. doi: 10.1093/nar/gkaa281 (PMC7261162; doi:10.1093/nar/gkaa281)
Supplement: gkaa281_Supplemental_File [file gkaa281_supplemental_file.pdf]

March 24, 2020

# High Specificity and High Spatial Restriction of Self-Biotinylation by DNA and RNA G-Quadruplexes Complexed with Heme.

Prince Kumar Lat[1], Kun Liu[2], Dev Nandan Kumar[1], Kenneth K. L. Wong[1], Esther M. Verheyen[1], and Dipankar Sen[1, 2] *

[1]Dept. of Molecular Biology and Biochemistry and [2]Dept. of Chemistry,

Simon Fraser University, Burnaby, British Columbia V5A 1S6, Canada

## SUPPORTING INFORMATION

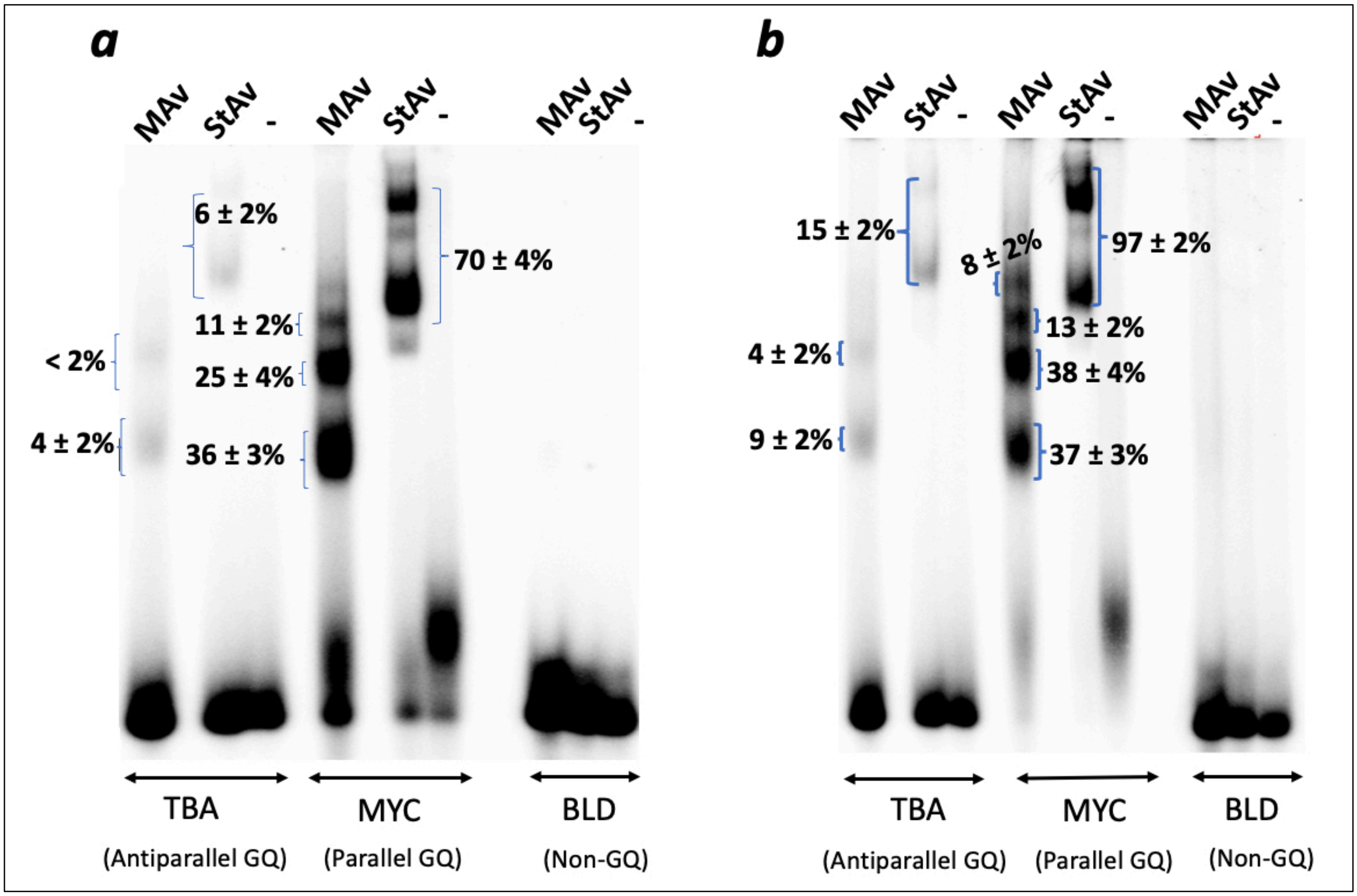


**Supporting Figure S1:** The consistency of GQ biotinylation levels in two independent experiments using the standard 5 µM heme (panel ***a***) and of biotinylation with a higher (50 µM) heme concentration (panel ***b***). For each of the above, two independent experiments were carried out and the error bars represent deviations from the mean. "MAv" refers to monoavidin, and "StAv" streptavidin. "-" above a gel lane indiates that neither streptavidin or monoavidin had been added to that DNA sample.

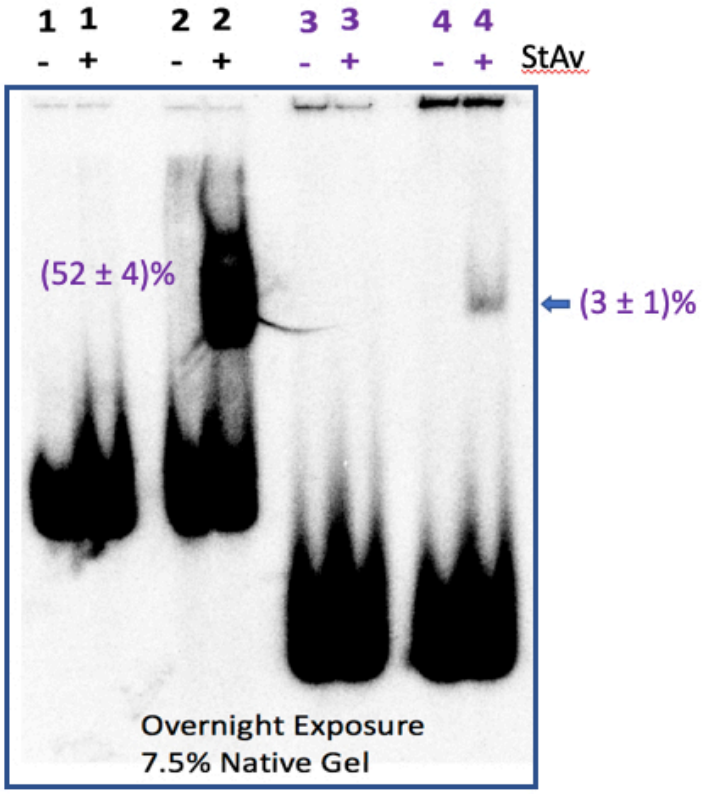


**Supporting Figure S2:** A longer exposure of the gel shown in **Figure 5** ***b***.

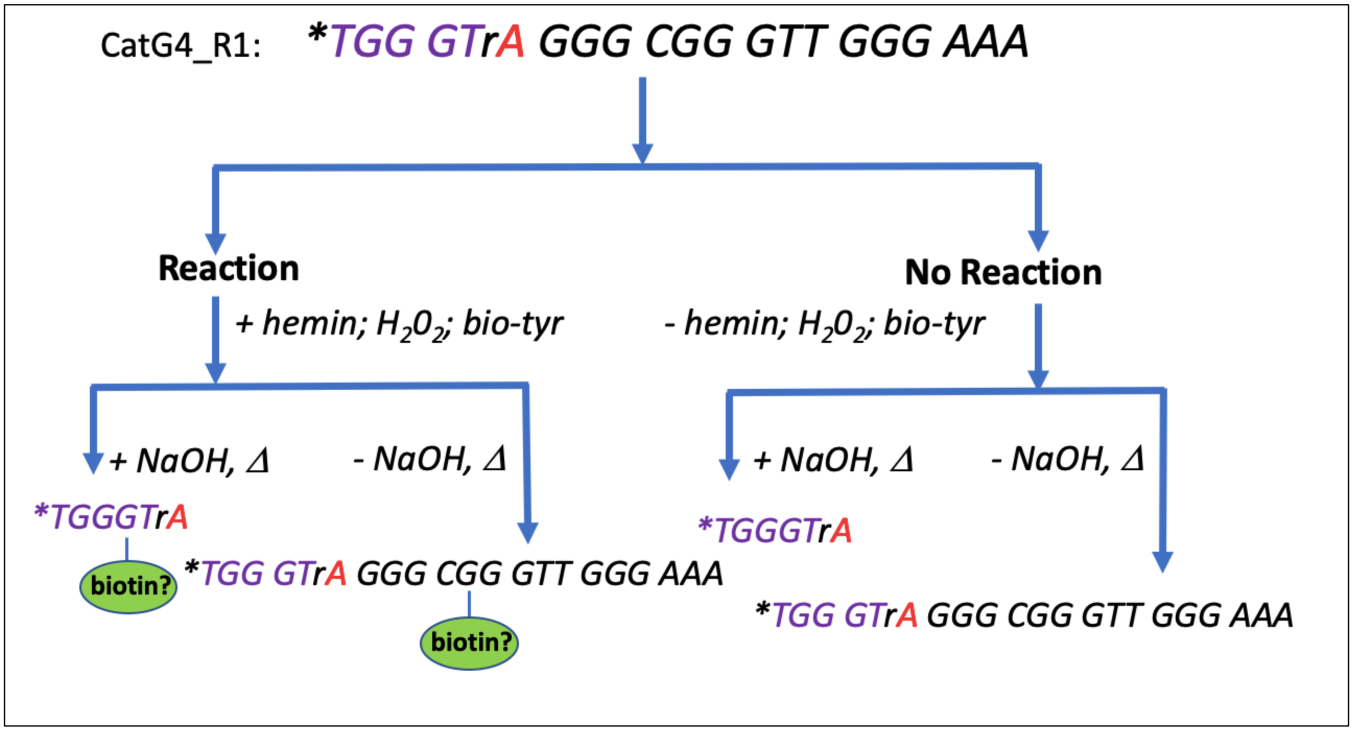


**Supporting Figure S3**: Schematic showing the experimental approach to determining the distribution of appended biotins in the GQ formed by "CatG4".

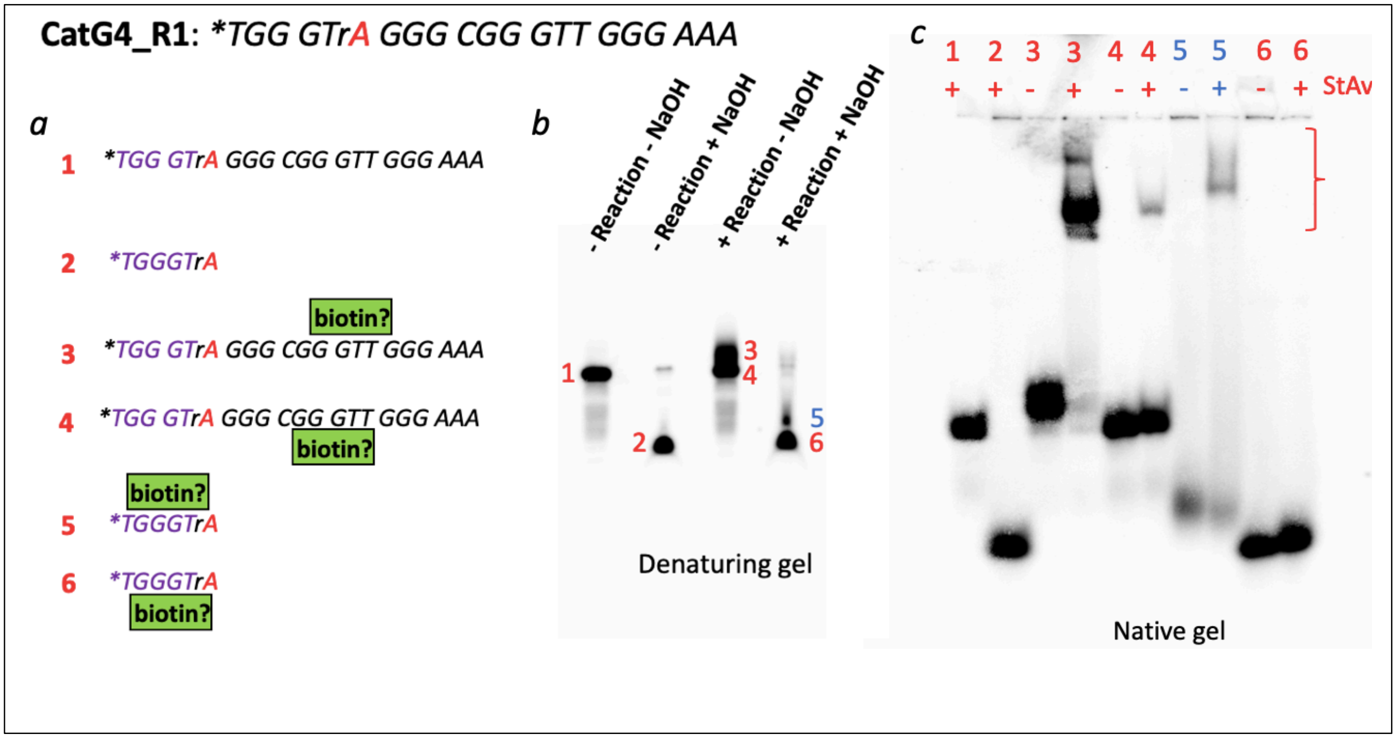


**Supporting Figure S4:** Determination of the extent of biotinylation within two component pieces (sequences 1 and 2 shown in ***a***) of the oligonucleotide "G4_R1" (or CatG4_R1). ***b***: A denaturing gel showing G4_R1 treated with hemin, BT, and $H_2O_2$ ("Reaction") or not treated, either cleaved in two by heating with NaOH (or not). The six $^{32}$P-labeled fragments, 1-6, were purified, and then mixed with soluble streptavidin (StAv). These were then run on a native gel (***c***). The red bracket in ***c*** shows the streptavidin-retarded bands.

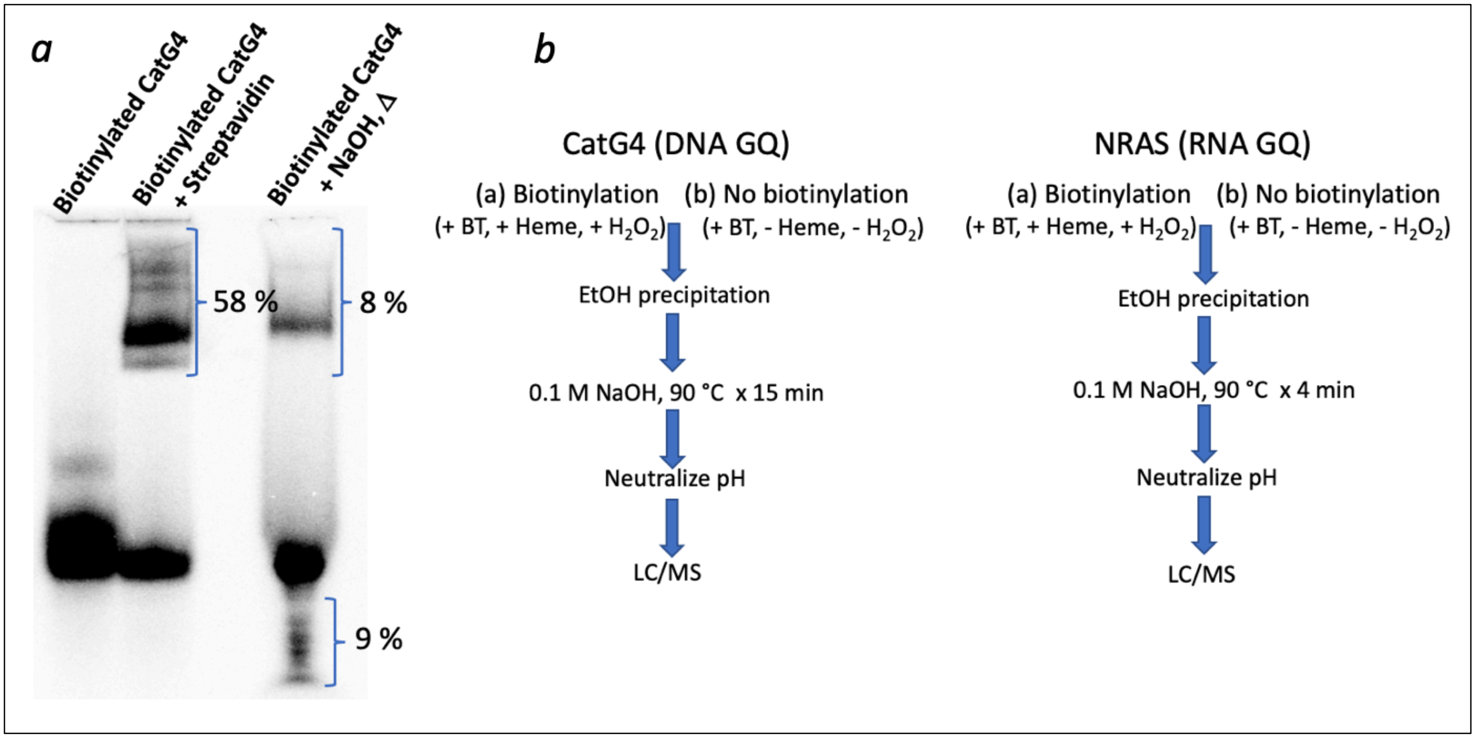


**Supporting Figure S5** ***a:*** Effect of 0.1 M NaOH and heating upon the biotinylated GQ, CatG4. Shown is a 7.5% native (non-denaturing) gel, whose first two lanes on the left show, respectively, biotinylated CatG4 (generated by the standard heme/$H_2O_2$/biotin-tyramide reaction) without and with streptavidin added prior to loading in the gel. From the second lane, it can be seen that 58% of the DNA has been biotinylated (retarded by streptavidin binding). Treatment with 0.1 M NaOH at 90°C (lane at far right), however, shows that such a treatment leaves only 8% of the DNA biotinylated. A portion of the DNA (9%) is also shown to be broken down in size by this treatment. ***b***: A reaction scheme for an LC/MS-based scheme for looking at whether biotin tyramide (or a related product) is in fact released from biotinylated DNA (CatG4) and RNA (NRAS) GQs following the hot base treatment. The MS data are shown in Supporting Figure S6.

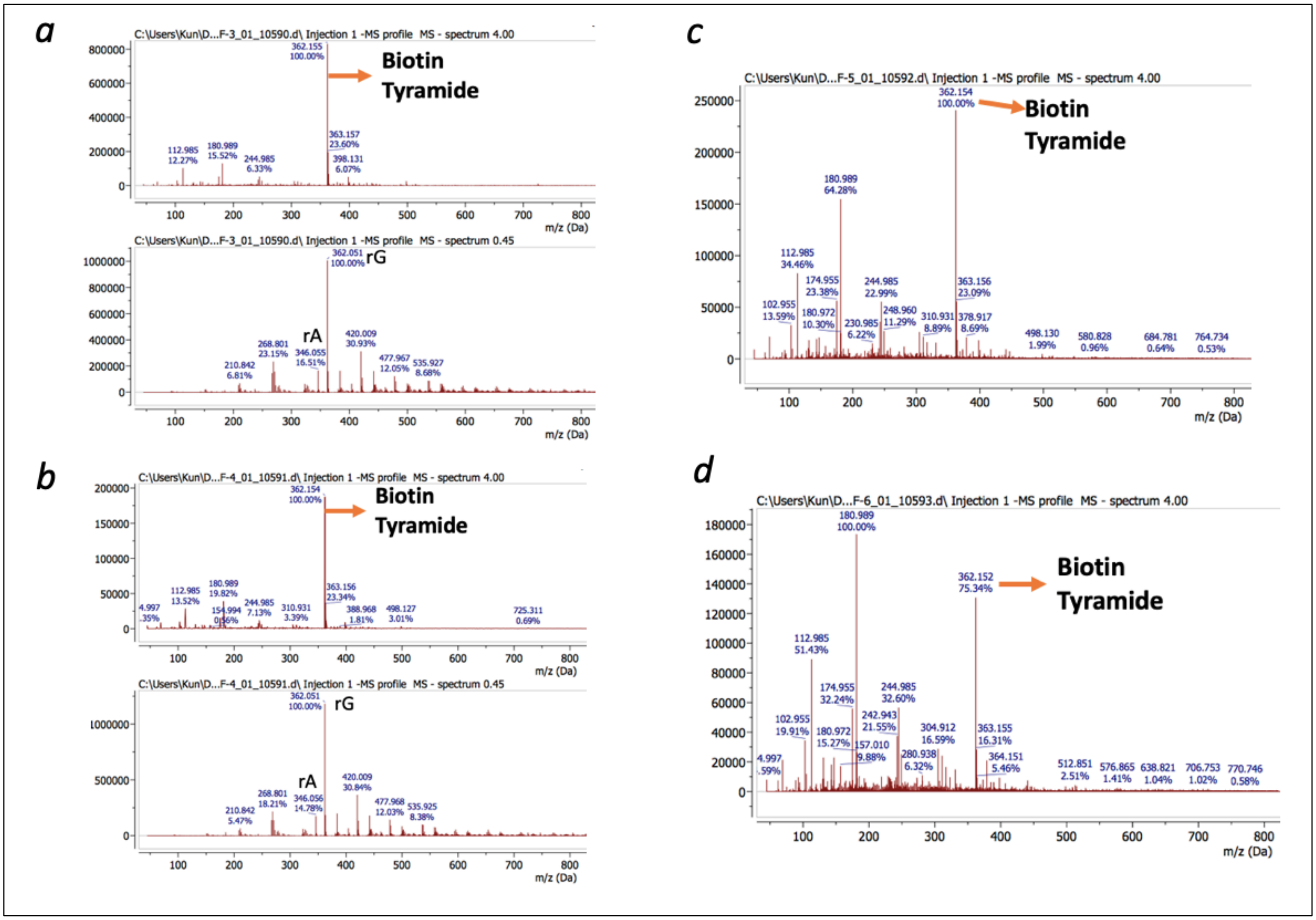


**Supporting Figure S6:** LC- ESI Mass spectrometry analysis of solutions (***a***) “NRAS RNA biotinylation”; (***b***) “NRAS RNA no biotinylation”; (***c***) “CatG4 DNA biotinylation”; (***d***); “CatG4 DNA no biotinylation”. ***a*** and ***b*** show both biotin-tyramide mass and abundances, as well as the mass and abundances of rGMP and rAMP generated from alkaline hydrolysis of the NRAS RNA (rCMP and rUMP are very small peaks). ***c*** and ***d*** show biotin-tyramide mass and abundances.

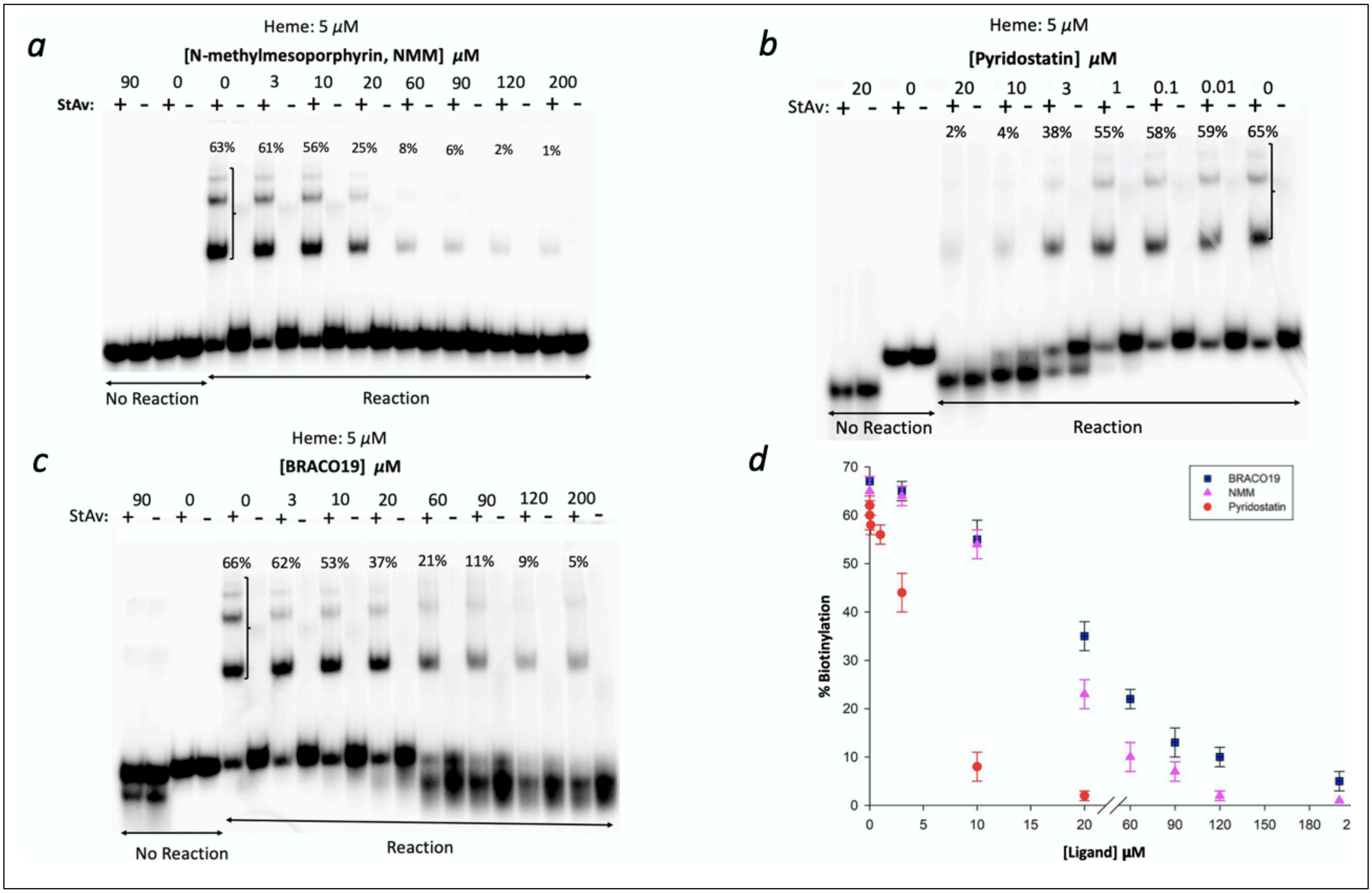


**Supporting Figure S7:** Impact of the titration of three non-heme GQ-binding ligands upon the GQ self-biotinylation reaction. Titration of 0-200 μM N-methylmesoporphyrin (NMM) (***a***); 0-20 μM Pyridostatin (***b***); and 0-200 μM BRACO19 (***c***) to 5 μM heme•"CatG4-ext" complex results in gradual abolition of GQ self-biotinylation. In all three panels, the bracket indicates streptavidin (StAv)-retarded DNA species. Panel ***d*** plots percent GQ biotinylation measured in response to concentration of added ligand (here the data represent quantitation of three independent experiments carried out with each of the GQ-binding ligands, NMM, Pyridostatin, and BRACO19. The error bars indicated represent one standard deviation from the mean.

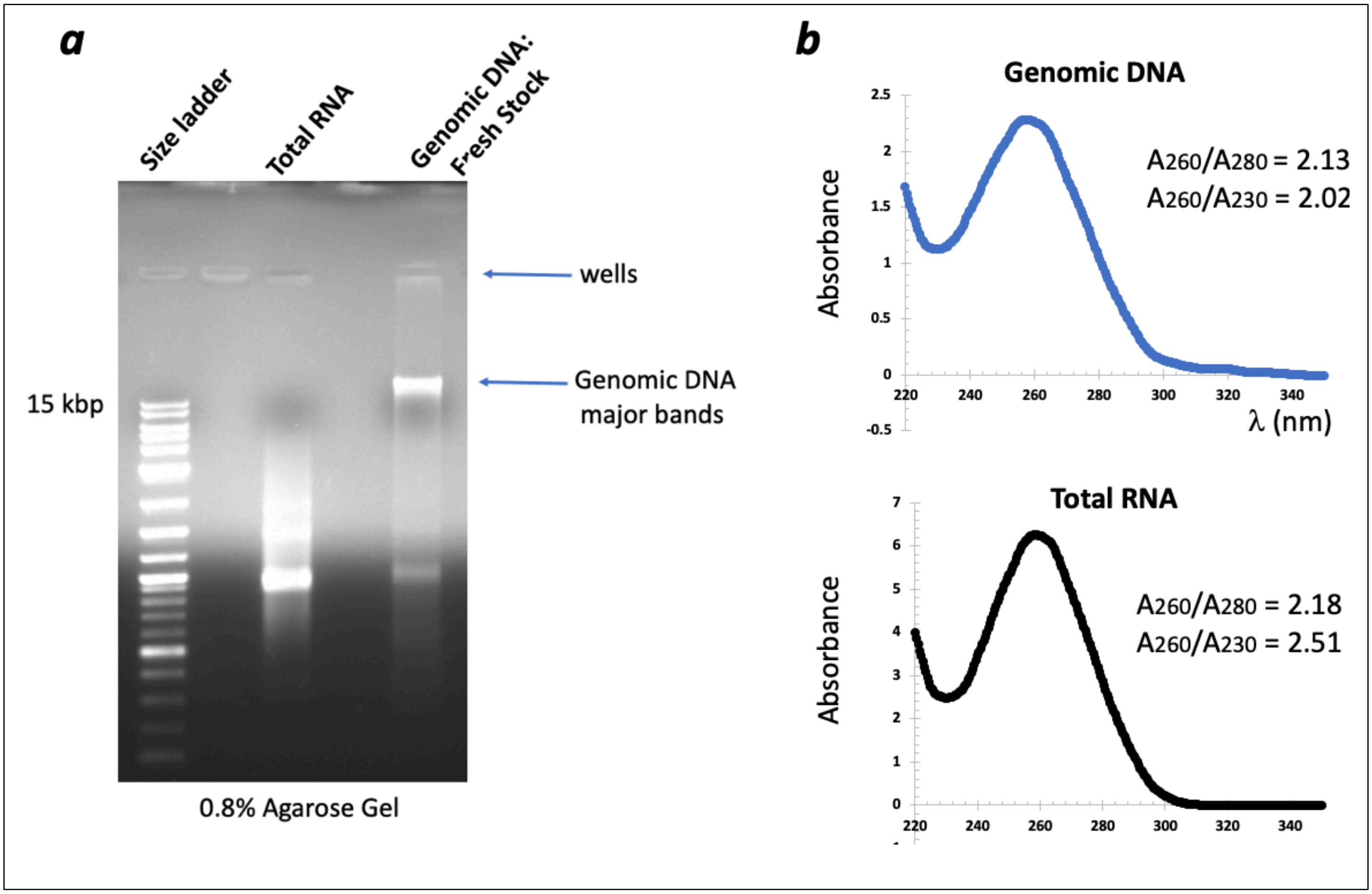


**Supporting Figure S8:** Examination of the purity of biotinylated DNA and RNA extracted using commercial kits from treated Drosophila embryo salivary glands. Panel ***a*:** A 0.8% agarose gel showing (left) total RNA and (right) genomic DNA purified from the glands. Panel ***b***: UV-Vis spectra of the purified total RNA and genomic DNA. The $A_{260}/A_{280}$ ratios, which are > 1.8, indicate high purities (free of proteins) for both DNA and RNA preparations.

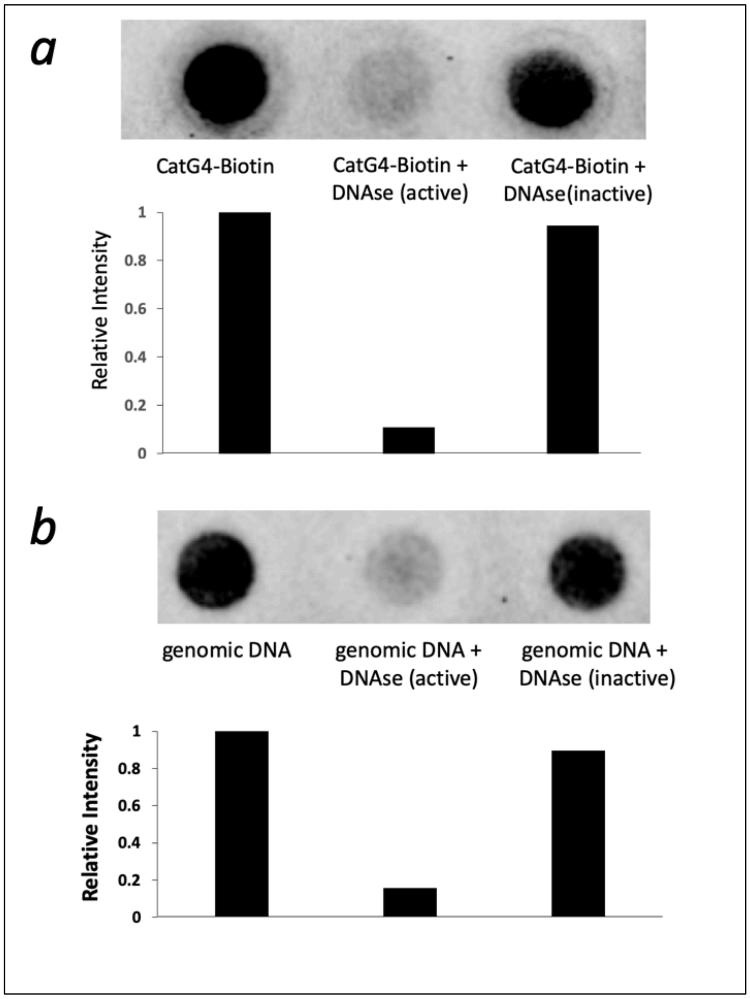


**Supporting Figure S9:** Evidence that the biotin detected, using streptavidin-HRP-generated chemiluminescence output signal, is from biotinylated DNA (and not biotinylated proteins) extracted from treated *Drosophila* embryonic salivary glands. Panel ***a***: A chemically biotinylated oligonucleotide (3'-biotinyl CatG4) was used as a positive control, spotted by itself (left); treated with DNAase I (centre); and with heat-deactivated DNAase I (right). Panel ***b***: the same analysis as in panel ***a***, except on total genomic DNA extracted from *Drosophila* 3$^{rd}$ instar larval salivary glands.

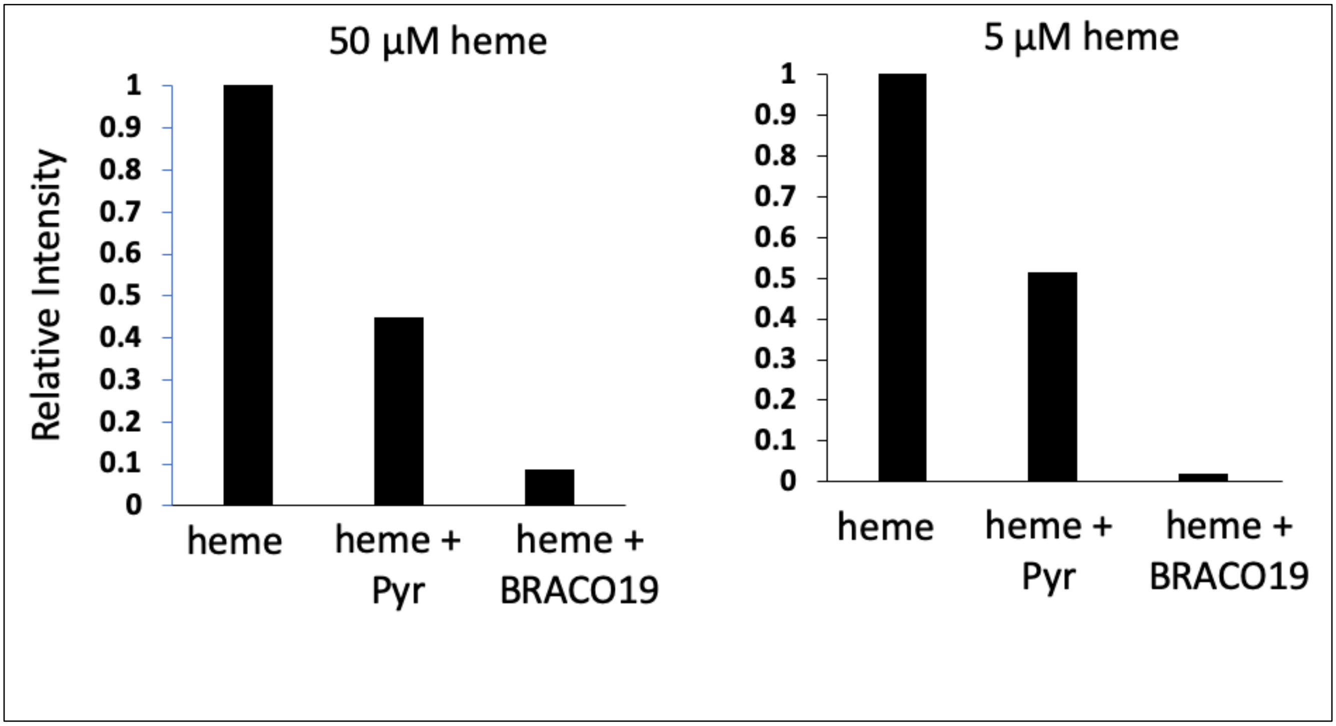


**Supporting Figure S10:** Quantiation of the chemiluminescence output data from the biotinylated total RNA blots shown in **Figure 9d**.
